# A Group of 500 Women Whose Health May Depart Notably From the Norm: Protocol for a Cross-Sectional Survey

**DOI:** 10.2196/resprot.7993

**Published:** 2017-11-23

**Authors:** Christoph Schnelle, Eunice J Minford, Vanessa McHardy, Jane Keep

**Affiliations:** ^1^ School of Public Health Faculty of Medicine University of Queensland Goonellabah Australia; ^2^ Faculty of Medicine, Health and Life Sciences Queen's University Belfast Antrim Ireland; ^3^ Light Education Training Ltd London United Kingdom; ^4^ The Leaders Leader Greater London United Kingdom

**Keywords:** women’s health, survey, public health, menstruation questionnaire, SF-36, ALSWH, Universal Medicine, preventive medicine, health care costs

## Abstract

**Background:**

Longitudinal studies of women’s health often seek to identify predictors of good health. Research has shown that following simple guidelines can halve women’s mortality. The ongoing Australian Longitudinal Study of Women’s Health (ALSWH) shows that Australian women are getting better at reducing their smoking and alcohol use, and are generally diligent about attending recommended health screenings, but are becoming less successful at dealing with obesity. There are communities of women who live unusually healthy lives (Rosetans, Seventh-Day Adventists, traditional Japanese women), but their lifestyles are unlikely to be adopted widely. Universal Medicine (UM) is a complementary-to-medicine approach that emphasizes personal empowerment and the importance of menstrual health symptoms.

**Objective:**

This survey investigates whether the approximately 500 women associated with UM exhibit health status significantly above the norm. As part of this investigation, questions for a newly developed menstrual attitudes questionnaire will also be evaluated.

**Methods:**

A quantitative cross-sectional survey of women in a UM cohort was designed with the help of three focus groups of women at three life stages: in menses, peri-menopausal, and menopausal. The menstrual attitudes portion of the survey incorporates the insights of these women regarding female health issues. The survey also includes 41 questions taken from the ALSWH. Focus groups generated additional questions about symptoms experienced and attitudes toward female health issues. ALSWH questions, including a range of health scales like the Short Form 36 (SF-36), Center for Epidemiologic Studies Depression Scale, Perceived Control Scale, Kessler Psychological Distress Scale, and the Multi-Item Summed Score for Perceived Stress, along with questions about experienced major health events, were investigated and incorporated if considered suitable. At the time of publication of this protocol, data collection has been completed.

**Results:**

The validity of the menstrual attitudes questionnaire will be evaluated with Cohen’s kappa. ALSWH respondents and UM participants will be compared, using unweighted regression or regression weighted or normalized by age, education, and interest in alternative treatments (to increase comparability), as appropriate. Analyses will determine whether UM-related variables (being a UM participant, length of UM participation, number of UM events attended) are associated with: differences in the number of major health events and health symptoms experienced; SF-36 physical and mental health scores; body mass index; and consumption of alcohol, tobacco, sugar, salt, caffeine, and dairy.

**Conclusions:**

If women in the UM cohort are truly in substantially better health than the norm, further investigations may be worthwhile to see whether UM plays a causal role, and whether the women’s practices are generalizable.

**International Registered Report Identifier (IRRID):**

DERR1-10.2196/7993

## Introduction

Various behavioral dimensions are strong predictors of health for both women and men. One study identified seven behaviors as being of health significance in a cohort of 6928 46-to-70-year-old people from Alameda county (near San Francisco, California) who were followed from 1965 to 1984: (1) not smoking; (2) using alcohol moderately (if at all); (3) exercising at least moderately; (4) sleeping 7 to 8 hours each 24-hours; (5) maintaining moderate weight; (6) eating regular meals; and (7) eating breakfast [1,2]. Respondents who reported performing six or seven of these behaviors had less than half the mortality, and those who performed four or five of the behaviors had approximately two-thirds the mortality, compared with those who performed fewer than three [1,2].

### Exemplary Populations

There are groups of people who have been documented as having unusually healthy profiles, including the inhabitants of the small American town of Roseto, Pennsylvania [3], Seventh-Day Adventists [4], Japanese living a traditional Japanese lifestyle [5], and senior Whitehall (United Kingdom) civil servants [6].

In Roseto, people were living according to traditional Italian roles, where residents tended to have very strong community ties. The diet of this population was nutritionally poor, and many did hard and dangerous work in a slate quarry, but their heart disease morbidity and mortality were much lower than those of inhabitants of surrounding towns [3]. Seventh-Day Adventists have lower coronary heart disease rates and lower rates of many cancers, among other health indicators, but they are a traditionalist Christian offshoot with strict rules [4]. Strict hierarchies were, and are, an important feature of Japanese life [7]. The better health of high-level British public servants may simply be evidence that those with better general habits are better able rise to higher positions [6]. Therefore, none of these four groups is without its limitations in terms of producing lifestyles that the general population would be likely to embrace.

### Women-Specific Research

A 1999 report that investigated the inclusion of women in various types of medical research found that in nongender-specific research, women were vastly underrepresented as research subjects [8]. Rodin and colleagues [9] have contributed significantly to the understanding of women’s health concerns, which differ from those of men. These researchers described women-specific health concerns such as hysterectomy, dysmenorrhea, caesarean section, almost all incidences of breast cancer, and some concerns not exclusively (but primarily) relevant to women, such as rheumatoid arthritis, lupus, osteoporosis, and eating disorders [9]. It has also been documented that 70% of psychoactive medications (eg, antidepressants, tranquilizers) are prescribed to women [10], and two-thirds of all surgeries are performed on women [11].

Measuring and improving female health is an important task for many governments, particularly those that have aging populations, and in which the incidence and prevalence of illness and disease are rising sharply. Unfortunately, this is a widespread problem: the rates of large-scale health issues like obesity and diabetes are rising [12,13]. The associated rise in health care costs adds to the urgency of this problem [14].

In Australia, the Australian Longitudinal Study on Women’s Health (ALSWH), with 58,000 women, was inaugurated in 1986 and added a further 17,000 women aged 18-23 years in 2012/2013. The purpose of the ALSWH is to, “clarify cause-and-effect relationships between women's health and a range of biological, psychological, social, and lifestyle factors” [15]; hence, this study’s data holds promise for elucidating predictors of good health in women.

### Weight and Obesity

An important predictor of health is being overweight or obese. A 2014 review [16] cited the ALSWH papers on weight. Among the findings, it was found that the proportion of obese individuals rises until the approximate age of 65 years and then falls [16]. Overweight women are significantly more likely to develop hypertension, heart disease, asthma, diabetes, depressive symptoms, and polycystic ovary syndrome, and to report hysterectomy [16]. Women who gained at least 5 kg over three years reported more menopausal symptoms [16]. The review further reports that individuals well over 60 years of age who are overweight have an increased chance of developing foot problems, arthritis, incontinence, declining physical functioning, acute health events, and stroke [16]. The exception to this pattern of disease susceptibility is osteoporosis, “because the increased load on the skeleton of high body mass index (BMI) individuals promotes higher bone mineral density” [17].

A potentially worrying point from the perspective of future population health was that in the 1973-1978 cohort, the 18-year-olds gained approximately 200 grams/year more weight than did the 23-year-olds [16]. In that 1973-1978 cohort, having a partner added 1.8 kg, and delivering a first baby added 4.0 kg over 10 years [16]. Lower levels of education, working full time, and having a mother with lower education were also associated with gaining weight [16].

The 1973-78 cohort will be approximately 8 kg heavier in middle age than the 1946-1951 cohort was. Consistent with the pattern documented by Gomersall et al [16], the Australian Bureau of Statistics reports that obesity in the population at large peaks at a 35% prevalence at age 65 and then drops to 25% at age 80 [18]. The absence of being overweight or obese is a predictor of good health, but currently the prevalence of being overweight or obese is increasing on a worldwide basis [13].

### Health and Behavior Change

There is a plethora of associations between adhering to health guidelines and better health outcomes, as described in a major ALSWH report [19]. The report shows that women appear to be able to reduce their intake of clearly harmful substances (eg, smoking, alcohol; whether the latter is beneficial in moderate doses or not) and their adherence to screenings is consistently high and stable, or rising [19]. However, in practice it seems currently impossible to improve people’s daily behaviors of eating less or exercising more on a population-wide level. In fact, the opposite seems to be the case with Australians: the percentage of overweight and obese adults is continuously increasing [18], which is a process that seems to be happening in other countries as well [13]. Interventions that empower women to address factors of diet, weight management, stress, and mood will likely be most effective in improving their health.

Good health practices are well known to medical science; the issue is less one of knowing *what* to do than knowing *how* to increase interest and action in adopting a healthy lifestyle overall among the wider population. For example, even diagnosis of a chronic disease (eg, heart disease, diabetes, asthma, breast cancer, arthritis, or depression) does not lead to improved levels of physical activity [20]. Such a diagnosis in itself is not generally adequate to induce women to improve their health behavior; although some women do make improvements, this gain is offset by those whose behavior actually worsens [20]. Counselling from doctors has been shown to work for high-risk patients [21], but is not being implemented on a level that would improve population health. In addition, adherence to doctors’ guidelines for chronic ailments is typically 50% or less [22]. A substantial group of women who are managing to adopt good health practices would therefore be of great interest to medical science and those responsible for public health, especially if the women’s lifestyle skills and habits are teachable and transferable. Our goal is to study participants in a program called Universal Medicine (UM), to determine whether they have health advantages over women at large.

### Universal Medicine

UM was founded in 1999 by Serge Benhayon, with the stated goal of, “providing Complementary Health & Healing Services that are Universal in their approach towards medicine and healing” [23]. Although Benhayon has no background in medicine or healing, as of 2015, approximately 200 men and 500 women were coming regularly to workshops and seeking treatments from UM-accredited practitioners. Benhayon describes UM’s mission as follows:

Through practical philosophies that inspire more self-caring and self-loving choices in daily life, Universal Medicine supports people to explore their overall well-being, the development of energetic awareness, and the depth they can bring to their quality of life and relationships [23]

UM teachings are delivered in the form of lectures, audio recordings, and treatments from UM clinics. Regular courses, workshops, and retreats are conducted throughout Australia and internationally. Individual participation levels range widely from consuming one or more webcasts per year to regular physical attendance at events in North Eastern New South Wales, Australia or in Frome, Somerset, United Kingdom, which receives approximately 700 regular visitors, including a number of health care professionals.

Over time the authors of this paper became aware of the fact that the large majority of participants in UM appear to lose weight with little or no effort and yet also report gains in vitality, as evidenced by observations that participants seem to be able to work or study for longer periods of time. These changes occur despite the fact that neither weight loss nor increased vitality is ever explicitly considered or targeted during UM events. Informal surveys have also indicated that very few members of the group eat gluten or dairy, drink alcohol or smoke, or consume caffeine. These individuals appear to have less added sugar and salt in their diets than the normal population does, and also seem to have little or no difficulty maintaining such a diet.

If these observations are accurate, and if the skills and behaviors associated with UM participants’ dietary choices and weight loss are transferable to the population at large, then UM holds a key to very substantial decreases in population health expenditure via reductions in obesity levels and decreased consumption of alcohol and tobacco. UM also differs from the norm in its attitude toward women’s health. The numerous and varied menstrual health issues that women encounter are not treated as nuisances to be managed or as illnesses to be cured, but as messages from the body that a change in attitude or lifestyle, or both, is needed. Such changes may be substantial at times, and do not supplant (but rather augment) visits to registered medical practitioners and adherence to prescribed treatments.

If this group of women is particularly skilled at managing menstrual health issues, and if these skills are transferable, then because menstrual symptoms like dysmenorrhea have prevalence rates as high as 90% [24] and menstrual symptoms are widespread [25,26], there would be further substantial population health benefits.

Serge Benhayon describes the lifestyle associated with UM as *The Way of the Livingness*, and *Livingness* is described as follows:

In a nutshell The Livingness is simply about living as our true selves... It is our ability to live and express who we truly are inside, and taking this into our day to day life. Everything we do say or think and every action we take or do not take contributes to our Livingness. Our choices, actions and inactions have a direct effect on our health, physical body, planet and each other. They can be the cause of our greatest healing or harm [27]

### What is Taught by Universal Medicine?

The core teaching of UM is that our bodies are a source of truth. An introductory course (Livingness 1) teaches how to *connect* to one’s body and to experience the consequences of that connection. The next course (Livingness 2) addresses emotions we have absorbed in the past. For example, a commonly used term is *feeling sick to our stomach* from emotions, and the extension of that everyday wisdom in UM is that some of the effects of emotions may linger much longer than the physical symptoms. The third course (Livingness 3) shows how to deal “with the sabotaging hindrances that prevent the real you from being in the fore” [28], showing that the quality in which everyday actions are performed can make a substantial difference to one’s well-being. Each of these courses can be completed in one day. In addition, UM presents training in its healing modalities [29], which consists of approximately 10 philosophical lectures in Australia annually, and three 5-day retreats annually.

### Possible Universal Medicine Effects

A medical doctor who treats a number of UM participants reported to one of the researchers (CS) that the participants are particularly able to take on a treatment plan and adhere to it. In addition, UM-affiliated women also appear more prone to adhere to health guidelines that are very similar to government-recommended ones. A visual gallery of the typical changes UM participants experience is available [30].

Hence, there is a possibility that the group is above average or even well above average in a substantial number of physical and mental health indicators. UM has developed a number of modalities that are complementary to medicine (ie, not developed within a mainstream medical environment). Evaluating these intervention techniques is beyond the scope of this survey; however, a randomized controlled cross-over trial of one of the UM modalities, chakra-puncture, is underway. A randomized controlled trial of another UM modality (esoteric connective tissue therapy) has received ethical approval from the University of Queensland, with a published protocol paper [31].

### Female Menstrual Symptoms

A further substantial, but under-investigated, area of women’s health is common menstrual symptoms. Approximately 80% of women in an ALSWH study [32] reported experiencing premenstrual syndrome, and 60% reported dysmenorrhea over the 12-year period in which members of the cohort were aged 22-27 years at the start and 34-39 years at the end. However, some women experienced increasing prevalence, others had decreasing prevalence, and some had constant high or low prevalence [32]. These findings suggest that there may be external or internal factors influencing these symptoms, and invites the possibility of changing these factors. For example, smoking increases menstrual symptoms and miscarriages, with odds ratios increasing with numbers of cigarettes smoked and younger age of smoking onset [33].

Women who have fewer menstrual symptoms or conditions have a reduced rate of hysterectomies [34]; hence, reducing these symptoms and conditions may have positive implications for the cost of health care. There is evidence that some female health issues are associated with choices women make in their lives. Herber-Gast et al [35] showed that both night sweats and hot flushes are positively associated with education, weight, smoking, drinking, premenstrual tension, diabetes status, and early age at first pregnancy. These associations were present whether or not the women were peri-menopausal or menopausal.

Women who are 45-50 years old experience physical declines, with those who are peri-menopausal or on hormone replacement therapy reporting substantially stronger declines (expressed in reduced SF-36 scores [36]) compared with those who remained premenopausal [37]. Therefore, a group of women whose physical health does not decline when their menstrual status changes, even after accounting for age, may be of interest, as might a group with a consistent drop in these symptoms.

### Objectives

The proposed survey will investigate whether women who participate in UM have a superior health profile compared with the respondents to the ALSWH. These data would allow a conclusion as to whether UM is worth investigating further to assess the presence of any causal links between specific aspects of the program and improved health.

## Methods

### Design

A quantitative cross-sectional survey design will be used to collect data via an online survey. No directly identifying data (name, address, or birthdate) will be collected; however, indirectly identifying data such as partial medical history and age in years will be collected. Hence, parts of the data will not be available for inclusion in the public repository. Age will be presented as a range, and medical history data will be excluded.

**Figure 1:**
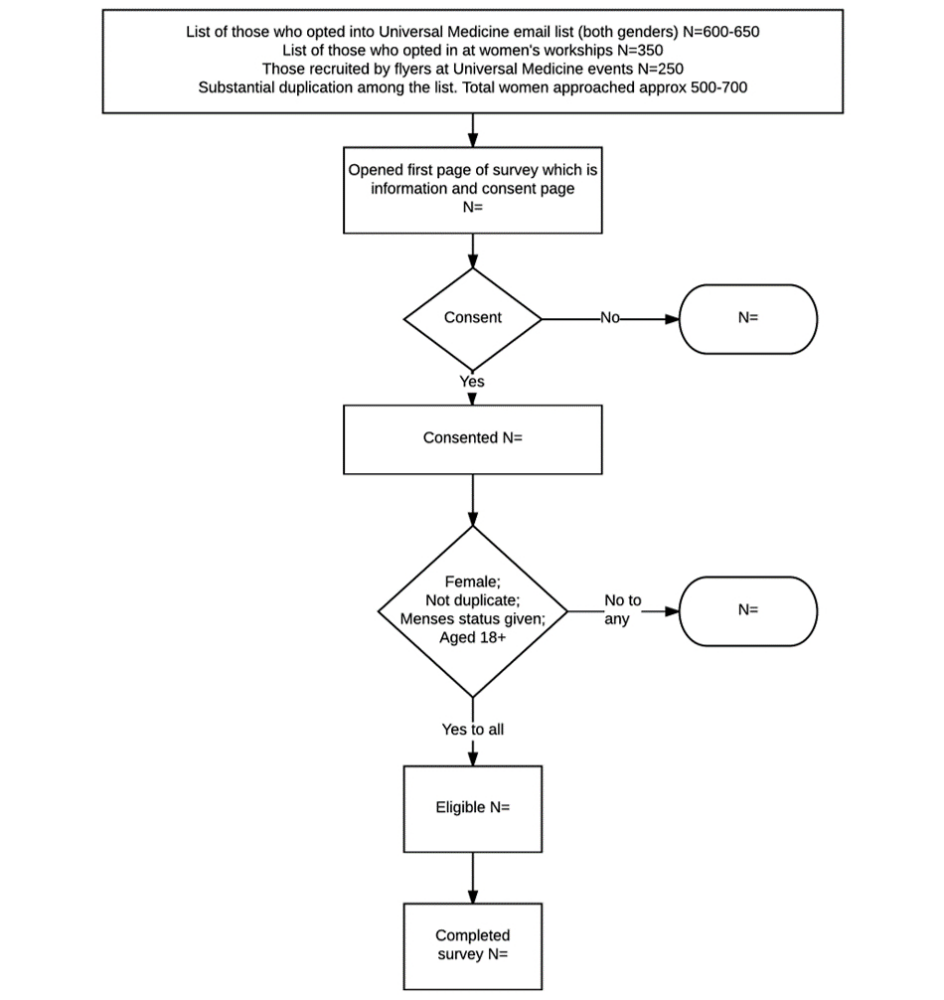
Recruitment flow diagram.

A recruitment flow diagram is presented in Figure 1. Recruitment will be undertaken by sending emails to a general information mailing list of approximately 650 members, of whom approximately 72% are female, who are targeted as regular UM events visitors. A second list of women who participated in UM-run women’s workshops (approximately 350 members with substantial overlap to the first email list) will also be used. In addition, visitors to several UM events will be given flyers with the same introductions given in the email circulars. These three methods are expected to reach over 90% of the target population. The same recruitment strategies used in previous surveys led to 482 and 393 respondents, representing 89% and 94% completion rates. The availability of the survey is to be approximately 7 weeks.

### Preliminary Focus Groups

As a starting point in developing the survey for the proposed study, two of the researchers (CS and VM) created three focus groups: (1) women in menses, (2) peri-menopausal women, and (3) menopausal women. Each group met four or five times via audio conferencing. The 15 participants were aged 18-72 years and were from Australia, the United Kingdom, Germany, and New Zealand. The education level of these women ranged from school-leaver to PhD and included a registered nurse, midwife and physiotherapist, an unregistered health practitioner, an exercise physiologist, and a university lecturer. Two members had a PhD. All 15 participants tested later versions of the survey.

Two menstrual attitude questionnaires [38,39] were investigated for possible use, but the tone of these questionnaires implies that menstrual health issues are problems to be minimized or avoided. This perspective did not agree with the experience of the women in the focus groups, who considered menstrual health issues useful, and thought that supportive signals from the body should be heeded; therefore, at the women’s suggestion, we decided to create a new questionnaire.

Focus group members read through the two existing menstrual attitude questionnaires and worked with the researchers to develop further questions based on recommendations from the researchers. Questions that achieved consensus were taken into the first draft and offered to the other focus groups as suggested questions for their part of the survey. This process was then repeated through 4 or 5 meetings, until the questions became stable. There was substantial overlap in questions among the three groups because, according to the focus groups, even women in their earlier menopausal years still have reproductive health issues. The developed questionnaire included objective items regarding physical and mental health symptoms and actions taken, and the participants’ attitudes toward their female health issues.

All three focus groups worked cooperatively to offer their opinions on which of the ALSWH questions to adopt to keep the survey to an acceptable length, while including as many ALSWH questions as possible. The focus was on questions that the researchers felt would yield the most relevant information about the respondents. One difficult issue was food, because the ALSWH questions are lengthy and were deemed not very relevant to the UM-affiliated respondents (eg, few UM respondents eat potatoes). Therefore, a short question about food items and their frequency of use and a question about current alcohol consumption were added. The focus group participants stated that they were in various stages of reducing their caffeine, added sugar, salty food, dairy, and alcohol consumption. The food question is designed to elicit which stage of reduction each participant is undertaking, if any. The question was adopted from a smaller biostatistics student project survey that had been developed with respondent feedback, so the food question was clearly understood by focus group members.

### Survey Development and Administration

This study emphasizes women’s health, and the survey will only be distributed to female UM respondents, who constitute over 70% of the UM population. The survey is comprised of two parts: (1) the newly developed menstrual attitudes questionnaire; and (2) the selection of questions commonly used in the ALSWH [40], to enable comparisons of the UM respondents to the ALSWH population.

The survey will be completed online using the *survs.com* survey platform [41]. Issues of a personal or sensitive nature are regularly and openly discussed at UM events, although anyone who opts out of any discussion is fully supported. To maintain this spirit of support, the survey was designed so that a respondent can skip any question that she does not wish to answer. The consent form at the beginning of the survey also makes it clear that the survey can be terminated at any time.

The ALSWH was chosen for comparison purposes with Australian women, as the estimated mean age of UM participants is the late 40s, and one of the ALSWH cohorts had their first particularly voluminous survey done in 1996, when the cohort’s ages ranged from 45-50 years. The second survey (in the 1998 ALSWH cohort) then filled in a number of gaps so that it was possible to extract the bulk of the comparative questions from those two surveys.

#### The Respondent Population

The respondent population is approximately 500-600 women who, as users of complementary medical services, may have had a similar profile to women who use alternative practitioners (ie, more likely to be middle-aged, have poorer health, and a higher usage of conventional medicine) [42]. On the basis of some researchers’ observations and informal surveys, the population’s average age is now in the high 40s, they have lost substantial weight since joining UM activities, and may have higher than average levels of vitality.

#### Final Composition of the Study Survey

After consultation with the focus groups, it was decided that all participants would answer the same selection of questions from the ALSWH, plus the newly developed question about food. Early versions of the survey were completed by individual members of the focus groups, and their feedback was incorporated into the survey. When the survey was nearing its final version, 10 of the focus group members completed it, and their feedback was also taken into account.

The first section of the survey asks for consent and confirms that the respondent is at least 18 years of age. Respondents are also asked about the date of their first UM event and their overall level of participation in UM. This section comprises 4 items. A major part of the survey for the proposed study is the SF-36 [36] questionnaire, which is also part of the ALSWH. The SF-36 is used to assess parameters of physical and mental health. In the proposed study, all items of the SF-36 will be used. Further ALSWH-employed scales used in the survey are questions from the Center for Epidemiologic Studies Depression Scale [43,44], Perceived Control Scale [45], Kessler Psychological Distress Scale [46], and the Multi-Item Summed Score for Perceived Stress [47].

On the menstrual attitudes section of the survey, menopausal women answer 42 questions, peri-menopausal women answer 54 questions, and women in menses answer 48 questions. Those who have never had any periods are invited to answer the menopausal women’s questions. Women self-select into the group that they think best fits their situation; however, if after choosing a group, they find that the questions do not appear to apply to them, they can back out of the survey and choose a different group.

The first set of questions consists of a list of symptoms with a response scale including *Yes*, *definitely/Yes*, *sometimes/No*, *not much/No*, and *not at all*, which is the same as that in the widely used Women’s Health Questionnaire [48]. This response scale was adopted after several more complicated response schemes were tried and discarded to reduce satisficing [49]. The survey contains 24, 26, and 26 menopausal, peri-menopausal, and menses symptom questions, respectively (of which 13 are common to all three groups). The survey also contains 24, 29, and 16 menstrual attitude questions, respectively (of which one is common to all three groups and seven are common to menopausal and peri-menopausal groups). The attitude questions use a 5-point Likert-type response scale ranging from “strongly disagree” to “strongly agree”. Multimedia Appendix 1 presents the derivations of the questionnaire items. A copy of the survey is provided in Multimedia Appendix 2.

The members of the focus group who tested the survey took approximately 75 minutes to complete it, but did not feel that this was excessive. The survey is similar in length to the ALSWH full surveys that are held once every 3 years. To make this study’s survey questions more comparable with those on the ALSWH, wherever possible the former have retained precisely the same wording as the latter, so the responses can be evaluated using the tools adopted by ALSWH-associated researchers [50].

At the time of publication of this protocol, data collection has been completed.

## Results

### Menstrual Attitudes Questionnaire

The validity and reliability of the developed questionnaire will be assessed using Cohen’s kappa [51-53]. Factor analyses for the questions common to all respondents will be used to ascertain the underlying factors for symptom questions. If the number of respondents is sufficient, the same will be attempted for the attitude questions and symptoms questions that are not common to all three menstrual statuses [54,55]. These preliminary validation steps may support the development of a draft menstrual attitudes questionnaire that can then undergo a full process of validation.

### Summary Analysis

Key indicators of health and comparisons of those indicators to ALSWH statistics (where available) that this survey aims to measure are: (1) SF-36 physical and mental health scores; (2) BMI; (3) symptoms experienced over the previous 12 months; (4) consumption of alcohol, tobacco, sugar, salt, caffeine, and dairy; and (5) prevalence and levels of depression, stress, distress, and perceived control, as measured by standard scales.

### Comparative and Detailed Analyses

Comparisons between UM participants and ALSWH respondents are planned, using both simple comparisons and comparisons weighted or normalized by age, education, and interest in alternative treatments (to increase comparability).

Regression analyses will be performed to determine whether SF-36 scores or BMI are associated with: (1) UM-related variables (length of UM participation, number of UM events); (2) demographic variables (age, age of menopause, age of menarche, education history); (3) menstruation-related attitudes and symptoms; (4) frequency of health-related symptoms; (5) major health events experienced; (6) alcohol and tobacco consumption; (7) sugar, salt, dairy, and caffeine consumption; (8) frequency of medical and other health practitioner visits; or (9) scores on other standardized scales (depression, perceived control, psychological distress, perceived stress), all of which are derived from the questions taken from the ALSWH questionnaires.

The expected sample sizes are relatively small (200-400 respondents per item, given that all items are optional), so any odds ratios and regression coefficients will be considered significant if their 95% confidence intervals exclude 1.0 (for odds ratios) or 0.0 (for regression coefficients).

## Discussion

An important issue is that this is the first attempt to collect scientific evidence about UM participants, and it is not even known whether the UM participants are more or less healthy (or both in different areas of health) than the general population. This survey will attempt to collect data to establish whether this group of people warrants further investigation. If the group is worth investigating scientifically, the survey could generate data that will allow the first steps of comparative effectiveness research [56,57] by testing for associations between health outcomes and other variables. A longitudinal study could then establish the sequence of events that lead to any associations. If these women do not spend substantially higher sums on health care than the general population but are markedly healthier, then this may be a pointer to insights that improve health care economics [58].

We have chosen to construct a survey that attempts to determine whether this group of women actually does experience exceptionally good health and, as a second step, to ascertain whether there are any connections between the attitudes of these women regarding women’s menstrual health issues and any aspects of their health status. The World Health Organization states that the focus on biological health for women in medical research led to a neglect of mental health research, with numerous negative consequences for the state of scientific knowledge [59]. This survey, with its coverage of physical and mental health and attitudes towards health, may allow research to see how these three areas relate to each other in this group of women.

### Issues with the SF-36 Instrument

The scoring instructions used by the ALSWH for the SF-36 will be employed [60]. The SF-36 physical and mental health summary scores are normalized to a mean of 50 and a standard deviation of 10. However, giving perfect answers to every question leads to a physical score on this questionnaire of only 56.5, and a mental score of only 62.5 (for this mental score one would, for example, need to tick *none of the time* in response to, “How often during the past four weeks did you feel tired?”).

A further SF-36 scoring feature is that to some extent, mental and physical health scores are mutually inhibiting. Physical health items are added as a negative score to the mental health score and mental health items are added as a negative score to the physical health score. This means that achievement of mental scores above 62.5 is only possible for people with less than perfect physical health and vice versa. The best theoretically possible mental health score can only be achieved by a person with very bad physical health and vice versa. This may affect the usefulness of the SF-36 if many respondents actually have a high level of both physical and mental health. One question is whether there will be a ceiling effect, with many scores near the surprisingly low upper limits of the SF-36.

The dose-response approach proposed here uses length of association and number of events or visits as the estimator of the UM *dose*. If results of the proposed study suggest that this group is worth investigating further, then in a longitudinal study, UM participants could be surveyed–perhaps in combination with objective health measures–at or near the beginning of their association with UM, and at designated follow-up intervals.

### Conclusions

If the surveyed women’s health is substantially better that the ALSWH cohort, if they are easily able to maintain that improved health, and if any of their practices can be translated in part or in whole to the general population, then investigating these women may be very valuable for obtaining insights into improving health and quality of life, and reducing the cost of health care in the general population.

### Ethics, Consent, and Permissions

Ethical approval CS23062015 was given by the School of Public Health Research Ethics Committee on June 23, 2015. The first question on the survey will obtain informed consent from all participants: “Do you give your consent to participate in this research survey?”, with response options, “Yes, I consent to participate in this survey” and, “No, I do not give consent.” Unless the respondent explicitly chooses *Yes*, the survey concludes at that moment.

### Availability of Data and Materials

Deidentified data will be made available. Such data will not include potential identifiers, as outlined in, “Preparing raw clinical data for publication: guidance for journal editors, authors, and peer reviewers” [61]. Specifically, this means that age (which will be categorized into intervals), lists of medical procedures undergone, and lists of major illnesses will be excluded. The data will most likely be stored with the Open Science Framework [62].

## Appendix

Multimedia Appendix 1 ALSWH scales to which UM participants respond, and the instruments from which the questions were derived.

Multimedia Appendix 2 Women in Livingness Survey.

## Abbreviations

ALSWH: Australian Longitudinal Study of Women’s Health
BMI: body mass index
SF-36: Short Form 36
UM: Universal Medicine

## References

[1] Breslow L (1998). Behavioral factors in the health status of urban
populations. J Urban Health.

[2] Guralnik JM, Kaplan GA (1989). Predictors of healthy aging: prospective evidence from the Alameda County
study. Am J Public Health.

[3] Egolf B, Lasker J, Wolf S, Potvin L (1992). The Roseto effect: a 50-year comparison of mortality rates. Am J Public Health.

[4] Fraser G (2003). Diet, life expectancy, and chronic disease: studies of Seventh-Day Adventists and
other vegetarians.

[5] Marmot MG, Syme SL, Kagan A, Kato H, Cohen JB, Belsky J (1975). Epidemiologic studies of coronary heart disease and stroke in Japanese men
living in Japan, Hawaii and California: prevalence of coronary and hypertensive heart
disease and associated risk factors. Am J Epidemiol.

[6] Marmot M, Smith GD, Stansfeld S, Patel C, North F, Head J, White I, Brunner E, Feeney A (1991). Health inequalities among British civil servants: the Whitehall II
study. Lancet.

[7] De Mente BL (2015). The role of harmony. Etiquette Guide to Japan: Know the rules that make the difference!.

[8] Mastroianni AC, Faden RR, Federman DD (1994). Women's participation in clinical research: from protectionism to
access. Women and health research: ethical and legal issues of including women in clinical
studies.

[9] Rodin J, Ickovics JR (1990). Women's health. Review and research agenda as we approach the 21st
century. Am Psychol.

[10] Ogur B (1986). Long day's journey into night: women and prescription drug
abuse. Women Health.

[11] Travis C (1988). Women and health psychology: biomedical issues.

[12] Smyth S, Heron A (2006). Diabetes and obesity: the twin epidemics. Nat Med.

[13] Malik VS, Willett WC, Hu FB (2013). Global obesity: trends, risk factors and policy
implications. Nat Rev Endocrinol.

[14] Goss J (2008). Projection of Australian health care expenditure by disease, 2003 to 2033.

[15] Australian Longitudinal Study of Women's Health (2016). Aims of the ALSWH.

[16] Gomersall SR, Dobson AJ, Brown WJ (2014). Weight gain, overweight, and obesity: determinants and health outcomes from
the Australian Longitudinal Study on Women's Health. Curr Obes Rep.

[17] Asomaning K, Bertone-Johnson ER, Nasca PC, Hooven F, Pekow PS (2006). The association between body mass index and osteoporosis in patients
referred for a bone mineral density examination. J Womens Health (Larchmt).

[18] (2013). 4338.0 - Profiles of Health, Australia, 2011-13.

[19] Dobson A, Byles J, Brown W, Mishra G, Loxton D, Hockey R, Powers J, Chojenta C, Hure A, Leigh L, Anderson A (2012). The Australian Longitudinal Study on Women's Health.

[20] Dontje ML, Krijnen WP, de Greef MH, Peeters GG, Stolk RP, van der Schans CP, Brown WJ (2016). Effect of diagnosis with a chronic disease on physical activity behavior in
middle-aged women. Prev Med.

[21] Hjermann I, Holme I, Byre KV, Leren P (1981). Effect of diet and smoking intervention on the incidence of coronary heart
disease. Lancet.

[22] Sabaté E (2003). Adherence to long-term therapies: evidence for action.

[23] Benhayon s (2016). Universal Medicine Home.

[24] Jamieson DJ, Steege JF (1996). The prevalence of dysmenorrhea, dyspareunia, pelvic pain, and irritable
bowel syndrome in primary care practices. Obstet Gynecol.

[25] Harlow SD, Campbell OM (2004). Epidemiology of menstrual disorders in developing countries: a systematic
review. BJOG.

[26] Dawood MY (2006). Primary dysmenorrhea: advances in pathogenesis and
management. Obstet Gynecol.

[27] Dalle Rive Carli G, Lessing N (2016). Livingness.

[28] Taylor JA (1953). A personality scale of manifest anxiety. J Abnorm Psychol.

[29] Universal Medicine Staff (2016). Universal Medicine Therapies.

[30] Universal Medicine Volunteers (2016). Presenting Universal Medicine.

[31] Schnelle C, Messerschmidt S, Minford EJ, Greenaway-Twist K, Szramka M, Masiorski M, Sheldrake M, Jones M (2017). Esoteric connective tissue therapy for chronic low back pain to reduce
pain, and improve functionality and general well-being compared with physiotherapy:
study protocol for a randomised controlled trial. Trials.

[32] Ju H, Jones M, Mishra GD (2014). Premenstrual syndrome and dysmenorrhea: symptom trajectories over 13 years
in young adults. Maturitas.

[33] Mishra GD, Dobson AJ, Schofield MJ (2000). Cigarette smoking, menstrual symptoms and miscarriage among young
women. Aust N Z J Public Health.

[34] Graham M, James EL, Keleher H (2008). Predictors of hysterectomy as a treatment for menstrual
symptoms. Womens Health Issues.

[35] Herber-Gast GM, Mishra GD, van der Schouw YT, Brown WJ, Dobson AJ (2013). Risk factors for night sweats and hot flushes in midlife: results from a
prospective cohort study. Menopause.

[36] Ware J, Kosinski M, Gandek B (2000). SF-36 health survey manual & interpretation guide.

[37] Mishra GD, Brown WJ, Dobson AJ (2003). Physical and mental health: changes during menopause
transition. Qual Life Res.

[38] Brooks-Gunn J, Ruble DN (1980). The menstrual attitude questionnaire. Psychosom Med.

[39] Moos RH (1968). The development of a menstrual distress questionnaire. Psychosom Med.

[40] Australian Longitudinal Study on Women's Health (2016). ALSWH.

[41] (2017). Survs.

[42] Adams J, Sibbritt DW, Easthope G, Young AF (2003). The profile of women who consult alternative health practitioners in
Australia. Med J Aust.

[43] Radloff LS (1977). The CES-D scale a self-report depression scale for research in the general
population. Appl Psychol Meas.

[44] Powers J, Young A, Russell A (2002). Australian Longitudinal Study on Womens Health.

[45] Lee J (2004). Data Dictionary Supplement, updated 24 March 2004.

[46] Kessler RC, Andrews G, Colpe LJ, Hiripi E, Mroczek DK, Normand SLT, Walters EE, Zaslavsky AM (2002). Short screening scales to monitor population prevalences and trends in
non-specific psychological distress. Psychol Med.

[47] Bell S, Lee C, Powers J, Ball J (2004). Data Dictionary Supplement 2001, updated December 2001.

[48] Hunter MS (2003). The Women's Health Questionnaire (WHQ): Frequently Asked Questions
(FAQ). Health Qual Life Outcomes.

[49] Krosnick J, Narayan S, Smith W (1996). Satisficing in surveys: initial evidence. New Dir Prog Eval.

[50] (2016). Australian Longitudinal Study on Womens Health.

[51] Reichenheim M (2004). Confidence intervals for the kappa statistic. Stata J.

[52] Wood J (2007). WebPsychEmpiricist Web Journal Internet.

[53] Cohen J (1960). A coefficient of agreement for nominal scales. Educ Psychol Meas.

[54] Thompson B, Brachresende D (2004). Exploratory and Confirmatory Factor Analysis: Understanding Concepts and
Applications.

[55] Torres-Reyna O (2010). Getting started in factor analysis (using Stata 10).

[56] Hirsch JA, Schaefer PW, Romero JM, Rabinov JD, Sanelli PC, Manchikanti L (2014). Comparative effectiveness research. AJNR Am J Neuroradiol.

[57] Sox HC, Greenfield S (2009). Comparative effectiveness research: a report from the Institute of
Medicine. Ann Intern Med.

[58] Drummond M, Sculpher M, Claxton K, Stoddart G, Torrance G (2015). Introduction to economic valuation. Methods for the economic evaluation of health care programmes.

[59] World Health Organization (2000). Women's mental health: an evidence based review.

[60] Russell A, Ball J, Spallek M (1998). Data Dictionary Supplement.

[61] Hrynaszkiewicz I, Norton ML, Vickers AJ, Altman DG (2010). Preparing raw clinical data for publication: guidance for journal editors,
authors, and peer reviewers. BMJ.

[62] (2017). Open Science Framework.

