# Supplementary material for: A Group of 500 Women Whose Health May Depart Notably From the Norm: Protocol for a Cross-Sectional Survey
Source: JMIR Res Protoc. 2017 Nov 23;6(11):e234. doi: 10.2196/resprot.7993 (PMC6173249; doi:10.2196/resprot.7993)
Supplement: Multimedia Appendix 1 [file resprot_v6i11e234_app1.pdf]

Multimedia Appendix 1. ALSWH scales to which UM participants respond, and the instruments from which the questions were derived.

**ALSWH Questions**
**Standardised questionnaires used**
Short Form 36 (SF-36)
Center for Epidemiologic Studies Depression Scale (CESD)
Perceived Control Scale
Kessler Psychological Distress Scale (K10)
Multi-item summed score for perceived stress [45]
**24 physical symptoms in the previous 12 months occurring ‘often’, ‘sometimes,’ ‘rarely’ or ‘never’**
Allergies, hay fever, sinusitis
Breathing difficulty
Indigestion / heartburn
Chest pain
Headaches / migraines
Severe tiredness
Stiff or painful joints
Back pain
Urine that burns or stings
Haemorrhoids (piles)
Other bowel problems
Vaginal irritation or unusual discharge
Hot flushes
Night sweats
Eyesight problems
Leaking urine
Mouth, teeth or gum problems
Avoided eating some foods because of problems with your teeth, mouth or dentures
Toothache
Hearing problems
Depression
Anxiety
Episodes of intense anxiety (e.g. panic attacks)
Palpitations (feeling that your heart is racing or fluttering in your chest)
**Other questions used in ALSWH**
Six questions about sleeping difficulties
Two questions about feeling rushed or having excess time
Height and weight, from which the BMI is calculated
Use of 11 weight loss methods at survey time
GP, specialist or hospital doctor visits
Ancillary and alternative medical practitioner visits and therapies undergone
Oral health
Alcohol usage

Tobacco consumption
Menstrual bleeding in the preceding 3 and 12 months
Increase or decrease in periods in the preceding 12 months
Time spent working and time spent on other activities
Abnormal pap test or abnormal mammogram history
Currently taking oral contraceptives or hormone replacement therapy
Age of first menstrual bleeding
Age of menopause
28 general health conditions like asthma, osteoporosis, cancer, mental illnesses
17 surgical or other major therapeutical procedures like chemotherapy or radiotherapy or hysterectomy
Weight loss of 5kg or more ever ‘on purpose’, ‘without effort, ‘for other reasons’
Weight gain of 5kg or more ‘ever’
Education history
**Non-ALSWH questions**
Whether the participants had caffeine, added sugar, added salt, dairy or alcohol, with responses ranging from “not for 5 years or more” to “more than once per day”. This is an original question and was selected because these items anecdotally have the highest variation in usage (sugar, salt, dairy, caffeine) and to highlight the expected extremely low level of usage (alcohol) by UM participants. The ALSWH food questions were not appropriate, as most of the foods would rarely or never be eaten by most UM participants.
Alcohol consumption at different life stages
Date of first UM event participation
Number of UM events or webcasts participating in annually
Menses status (‘menopausal’, ‘peri-menopausal’, ‘having periods’, ‘never had periods’)

Menstrual attitude survey questions

**Menstrual Attitude Survey Questions, Part I**
**Symptom questions**<br>**(Yes, definitely / Yes, sometimes / No, not much / No, not at all)**
**Common for all menstrual states**
A desire to have more order in my life [included as one of the questions in the menstrual attitude questionnaire: “(Do you feel a need to) keep things neat; orderly?”]
Abdominal cramps
Feeling emotional or sad
Endometriosis
Exhaustion
Feeling bloated
Feeling dizzy or faint
Food or drink cravings
Nausea or vomiting
Pre-menstrual (full moon) tension, but few people notice
Pre-menstrual (full moon) tension, noticed by many

Very sensitive, tender or sore breasts
Weight gain
**Menopause**
Being more forgetful at times
Difficulty concentrating
Feeling anxious
Feeling uncomfortable in the presence of others more frequently
Backaches
Bursts of energy
Headaches
Hot flushes
Mood swings – rapid changes in how you feel
Ovarian pain or cramps
Pimples, dry skin, greasy skin, erratic skin in general
Sudden sweats during the day
Tiredness, worse than in the past
**Peri-menopause**
Feeling relief or appreciation when my periods arrive
Feeling upset or disappointed when my periods arrive
Heavy periods
Heavy periods that are worse than before
Heavy periods that last longer than before
Hot flushes
Irregular period cycles
Normal periods that last longer than before
Ovarian pain or cramps
Painful periods
Period cycles that are substantially longer than in the past
Sudden sweats during the day
Tiredness, worse than in the past
**Menses**
Constipation and other unusual bowel movements
Feeling bothered or inconvenienced by my periods
Feeling controlled by my periods
Heavy clotting, slight clotting
Heavy periods
Intense irritation with others
Painful periods
Severe tiredness
Skipped periods
Swollen breasts
Tired or aching legs

**Menstrual Attitude Survey Questions, Part II**

Attitude questions (5-point Likert scale)
**Common for all menstrual states**
I feel I am at the mercy of the hormonal changes in my body.
**Menopause**
I am glad my periods are finished.
I am nurturing myself more.
I changed my life to a more harmonious pace.
I feel much wiser.
I feel my body is calling me to be more still.
I feel my body is calling me to be more tender.
I feel steadier than before.
I feel that my moods and my body are affected by the moon cycle.
I feel worthless since I don't have periods anymore.
I take more supplements than before.
Losing my periods gives me a feeling of loss and grief.
Menopause is a period of grace, giving me time to explore a new role in my life.
My body shape has changed in a good way.
My daily choices affect my bodily symptoms more strongly than in the past.
Since I have no periods anymore, I am not seen anymore.
**Peri-menopause**
I am nurturing myself more.
I changed my life to a more harmonious pace.
I feel embarrassed when I suddenly sweat or have hot flushes.
I feel much wiser.
I feel my body is calling me to be more still.
I feel my body is calling me to be more tender.
I feel quite unsettled because my cycle is so irregular.
I feel steadier than before.
I find my body is more easily affected by my daily living choices than in the past.
I find peri-menopause very upsetting.
I take more supplements than before.
I will be relieved or happy when my period stops.
I wonder if I am still of worth once I don't have periods anymore.
Losing my periods gives me a feeling of loss and grief.
My body shape has changed in a good way.
Once I have no periods anymore I won't be seen anymore.
Peri-menopause is a period of grace, giving me time to adjust to my new role in the world.
**Menses**
I am more tired during my period.
I dislike having periods.
I feel a need to be more gentle with myself when menstruating.
I feel at the mercy of the hormonal cycles in my body.
I notice how much my body supports me in the timing of when my periods arrive[1].
I notice how my emotions change during my cycle.

I notice how period pain, tender breasts, pimples or food cravings are related to how I am living.
I often experience a pressure or build-up before my period and then relief when my period arrives.
I pay little attention to my period cycle; it just comes and goes.
I talk with other women about my period comfortably.
I use painkillers to stop period pain so I can get on with my day.
My periods can come at very inconvenient times.
Periods are a good sign of your general health.
Periods are a sign of womanhood.

**Examples of ALSWH questions not included** These questions are listed to give an overview about questions considered off-topic or not of sufficient priority to include in the 75 minute time limit for the UM survey.
Detailed questions about GP (doctor) visits.
Various questions about ease of access to health care.
Do you have private health insurance?
During the past four weeks, how many different types of medication (eg tablets/ medicine), prescribed or over the counter have you used?
During the past four weeks have you taken any medications for nerves, sleep, depression, chronic condition?
Detailed questions about smoking and alcohol.
How much would you like to weigh?
Last week’s physical activity.
Questions about paid work and income.
What kind of transport are you using?
Have you ever experienced any form of physical, mental, emotional or sexual abuse or violence, either as a child, in an adult relationship, or at any other time?
Questions about living arrangements.
Questions about loneliness.
How old were you when your parents died?
Date of birth, postcode, study and marital status, home owner status, self assigned socio-economic class status.
Satisfaction with various aspects of life.

1 The women observed that in the past their periods sometimes appeared at very inconvenient times, and now at times that are much more convenient, like Saturday morning.]
