# Supplementary material for: A Group of 500 Women Whose Health May Depart Notably From the Norm: Protocol for a Cross-Sectional Survey
Source: JMIR Res Protoc. 2017 Nov 23;6(11):e234. doi: 10.2196/resprot.7993 (PMC6173249; doi:10.2196/resprot.7993)

Women in Livingness Survey

This survey is for women who are students of The Way of The Livingness.

We would like to know if there have been any differences for you in your life.

This survey is very helpful to see what changes the female students of the livingness are going through.

The survey takes about 45 minutes to an hour but can be interrupted and resumed at any time provided it is done on the same computer/tablet/phone and the same internet browser.

Your participation is entirely voluntary. You do not have to answer any questions and you can stop or withdraw from the survey at any time and any withdrawal will not affect your relationship with the researchers or Universal Medicine.

The question below asks about you giving your consent to participating in this survey.

This survey is for women who are students of the livingness, i.e. who regularly visit or participate in Universal Medicine events.

The purpose of the survey is to identify details in relation to your current health and your health at the time of your first Universal Medicine event. The survey is anonymous and completely voluntary, no name, date of birth or email details are requested. Neither the researchers nor Universal Medicine will know whether you participated or not.

Providing details of your health and well-being will allow the researchers, Vanessa McHardy and Christoph Schnelle to find out whether women who are students of The Livingness (which in this case means women who come to or participate in Universal Medicine events) have a level of health and well-being that is different from the general population and whether it was different from the general population at the time of your first Universal Medicine event.

In other words:

Are people who come to Universal Medicine events different from everybody else in their health and well-being now? Were they different when they came for the first time?

This survey will give you a chance to reflect and see if there have been any changes for the better or for the worse for you as an individual. Once we have collected and processed the data we will also be able to provide a report of the general result.

We do not expect you to get in any way upset, stressed or distressed during this survey. Should this happen anyway, please feel free to terminate this survey at any time for this or any other reason and or to email Christoph Schnelle cs@inyourinterest.com.au or Vanessa McHardy vmhediting@gmail.com advising him or her of what happened to you or if you have any further questions or feedback and we will take whatever action is necessary.

There will be a report of this study which will be shared among all those who received the email inviting them to this survey.

This study adheres to the Guidelines of the ethical review process of The University of Queensland and the National Statement on Ethical Conduct in Human Research. Whilst you are free to discuss your participation in this study with project staff (contactable on 02 6624 4242, country code 61 for Australia), if you would like to speak to an officer of the University not involved in the study, you may contact the Ethics Coordinator on 07 3365 3924 (country code 61).

1. Do you give your consent to participating in this research survey?

- ◯ Yes, I consent to participating in this survey
- ◯ No, I do not give consent

2. Could you tell us whether you are male or female?

◯ Female ◯ Male

Male Students of the Livingness

At the moment we are doing this survey only for women. We will prepare a version for men in the future.
However, it would be very useful for us if you answer the following questions.

3. If you remember the year, the month and or the day you attended your first Universal Medicine event or were introduced to The Livingness, could you enter it here? If you only remember the Year and or Month that would be very helpful as well.
If you don't remember, simply skip this question

Year

Month

Day

4. How often do you visit Universal Medicine events? *

- Once a year or less
- 2 - 5 times a year
- 6 - 10 times a year
- More than 10 times a year

5. If you remember the year, the month, and or the day you attended your first Universal Medicine event or were introduced to The Livingness, could you enter it here? If you only remember the Year and or Month that would be very helpful as well.

The year is particularly important.

For the year, examples are 2005 *or* 2014*, for the month, enter for example* 4 *for April or* 8 *for August*

Year

Month

Day

6. Could you give your age in years? *

7. How often do you visit Universal Medicine events or participate in webcasts? *

- Once a year or less
- 2 - 5 times a year
- 6 - 10 times a year
- More than 10 times a year

8. Which of the following fits you best? *

- I have not yet had a period
- I have periods and menopause will be at some time in the future
- Perimenopause: I am in the period leading up to menopause
- I am past my menopause

No period yet

9. If you are 18 or over, you are invited to answer the same questions as the menopausal women. The early questions will not fit you entirely, though the later questions will.

If you are under 18 this survey will end. *

- I am 18 or over
- I am under 18

Menopause

10. Menopause: **In the 12 months before you attended your first Universal Medicine event** and or were introduced to The Livingness, did you have any of the following

*Tick all that apply - **if you don't tick a row we assume that you didn't have that symptom.***

	Yes, definitely	Yes, sometimes	No, not much	No, not at all
Ovarian Pain or Cramps	☐	☐	☐	☐
Abdominal Cramps	☐	☐	☐	☐
Endometriosis	☐	☐	☐	☐
Very sensitive, tender or sore breasts	☐	☐	☐	☐
Tiredness, worse than in the past	☐	☐	☐	☐
Exhaustion	☐	☐	☐	☐
A desire to have more order in my life	☐	☐	☐	☐
Pre-full moon tension but few people notice	☐	☐	☐	☐
Pre-full moon tension noticed by many	☐	☐	☐	☐
Feeling bloated	☐	☐	☐	☐
Hot flushes	☐	☐	☐	☐
Sudden sweats during the day	☐	☐	☐	☐
Weight gain	☐	☐	☐	☐
Emotional or sad	☐	☐	☐	☐
Feeling dizzy or faint	☐	☐	☐	☐

11. **In the last 12 months** did you have any of the following:
('the last 12 months' means the 12 months ending today)
If you attended your first Universal Medicine event less than 12 months ago, did you have any of the following since you attended your first Universal Medicine event and or since you were introduced to The Livingness:

Tick all that apply - if you don't tick a row we assume that you didn't have that symptom.

	Yes, definitely	Yes, sometimes	No, not much	No, not at all
Ovarian Pain or Cramps	☐	☐	☐	☐
Abdominal Cramps	☐	☐	☐	☐
Endometriosis	☐	☐	☐	☐
Very sensitive, tender or sore breasts	☐	☐	☐	☐
Tiredness, worse than in the past	☐	☐	☐	☐
Exhaustion	☐	☐	☐	☐
A desire to have more order in my life	☐	☐	☐	☐
Pre-full moon tension but few people notice	☐	☐	☐	☐
Pre-full moon tension noticed by many	☐	☐	☐	☐
Feeling bloated	☐	☐	☐	☐
Hot flushes	☐	☐	☐	☐
Sudden sweats during the day	☐	☐	☐	☐
Weight gain	☐	☐	☐	☐
Emotional or sad	☐	☐	☐	☐
Feeling dizzy or faint	☐	☐	☐	☐

12. Part 2 of 2 Menopause: **In the 12 months before you attended your first Universal Medicine event** and or were introduced to The Livingness, did

you have any of the following:

Tick all that apply - if you don't tick a row we assume that you didn't have that symptom.

	Yes, definitely	Yes, sometimes	No, not much	No, not at all
Food or drink cravings	☐	☐	☐	☐
Nausea or vomiting	☐	☐	☐	☐
Feeling uncomfortable in the presence of others more frequently	☐	☐	☐	☐
Difficulty concentrating	☐	☐	☐	☐
Being more forgetful at times	☐	☐	☐	☐
Feeling anxious	☐	☐	☐	☐
Having bursts of energy	☐	☐	☐	☐
Having back aches	☐	☐	☐	☐
Mood swings - rapid changes in how you feel	☐	☐	☐	☐
Headaches	☐	☐	☐	☐
Pimples, dry skin, greasy skin, erratic skin in general	☐	☐	☐	☐

13. **In the last 12 months** did you have any of the following:
('the last 12 months' means the 12 months ending today)
If you had your first Universal Medicine event less than 12 months ago, did you have any of the following since you attended your first Universal Medicine event or were introduced to The Livingness:

Tick all that apply - if you don't tick a row we assume that you didn't have that symptom.

	Yes, definitely	Yes, sometimes	No, not much	No, not at all
Food or drink cravings	☐	☐	☐	☐
Nausea or vomiting	☐	☐	☐	☐
Feeling uncomfortable in the presence of others more frequently	☐	☐	☐	☐
Difficulty concentrating	☐	☐	☐	☐
Being more forgetful at times	☐	☐	☐	☐
Feeling anxious	☐	☐	☐	☐
Having bursts of energy	☐	☐	☐	☐
Having back aches	☐	☐	☐	☐
Mood swings - rapid changes in how you feel	☐	☐	☐	☐
Headaches	☐	☐	☐	☐
Pimples, dry skin, greasy skin, erratic skin in general	☐	☐	☐	☐

14. How do you currently feel about the following statements?

	Strongly disagree	Disagree	Neither agree or disagree	Agree	Strongly agree
My daily choices affect my bodily symptoms more strongly than in the past	○	○	○	○	○
I feel I am at the mercy of the hormonal changes in my body	○	○	○	○	○
I feel worthless since I don't have periods any more	○	○	○	○	○
Since I have no periods any more I am not seen any more	○	○	○	○	○
Losing my periods gives me a feeling of loss and grief	○	○	○	○	○
I am glad my periods are finished	○	○	○	○	○
Menopause is a period of grace giving me time to explore a new role in my life	○	○	○	○	○

I feel my body is calling me to be more still	◯	◯	◯	◯	◯
I feel my body is calling me to be more tender	◯	◯	◯	◯	◯
I take more supplements than before	◯	◯	◯	◯	◯
I feel steadier than before	◯	◯	◯	◯	◯
I am nurturing myself more	◯	◯	◯	◯	◯
I changed my life to a more harmonious pace	◯	◯	◯	◯	◯
My body shape has changed in a good way	◯	◯	◯	◯	◯
I feel much wiser	◯	◯	◯	◯	◯
I feel that my moods and my body are affected by the moon cycle	◯	◯	◯	◯	◯

15. How **did you feel** about the following statements **at the time you attended your first Universal Medicine event** and or were introduced to The Livingness?

	Strongly disagree	Disagree	Neither agree or disagree	Agree	Strongly agree
My daily choices affect my bodily symptoms more strongly than in the past	◯	◯	◯	◯	◯
I feel I am at the mercy of the hormonal changes in my body	◯	◯	◯	◯	◯
I feel worthless since I don't have periods any more	◯	◯	◯	◯	◯
Since I have no periods any more I am not seen any more	◯	◯	◯	◯	◯
Losing my periods gives me a feeling of loss and grief	◯	◯	◯	◯	◯
I am glad my periods are finished	◯	◯	◯	◯	◯
Menopause is a period of grace giving me time to explore a new role in my life	◯	◯	◯	◯	◯
I feel my body is calling me to be more still	◯	◯	◯	◯	◯
I feel my body is calling me to be more tender	◯	◯	◯	◯	◯
I take more supplements than before	◯	◯	◯	◯	◯
I feel steadier than before	◯	◯	◯	◯	◯
I am nurturing myself more	◯	◯	◯	◯	◯
I changed my life to a more harmonious pace	◯	◯	◯	◯	◯
My body shape has changed in a good way	◯	◯	◯	◯	◯
I feel much wiser	◯	◯	◯	◯	◯
I feel that my moods and my body are affected by the moon cycle	◯	◯	◯	◯	◯

Perimenopause

16. Perimenopause: **In the 12 months before you attended your first Universal Medicine event** and or were introduced to The Livingness, did you have any of the following:

*Tick all that apply - **if you don't tick a row we assume that you didn't have that symptom.***

	Yes, definitely	Yes, sometimes	No, not much	No, not at all
Ovarian Pain or Cramps	☐	☐	☐	☐
Abdominal Cramps	☐	☐	☐	☐
Endometriosis	☐	☐	☐	☐
Painful periods	☐	☐	☐	☐
Heavy periods	☐	☐	☐	☐
Heavy periods that are worse than before	☐	☐	☐	☐
Heavy periods that last longer than before	☐	☐	☐	☐

Normal periods that last longer than before	☐	☐	☐	☐
Period cycles that are substantially longer than in the past	☐	☐	☐	☐
Irregular period cycles	☐	☐	☐	☐
Very sensitive, tender or sore breasts	☐	☐	☐	☐
Tiredness, worse than in the past	☐	☐	☐	☐
Exhaustion	☐	☐	☐	☐

17. **In the last 12 months** did you have any of the following:
('the last 12 months' means the 12 months ending today)
If you attended your first Universal Medicine event less than 12 months ago, did you have any of the following since you attended your first Universal Medicine event or were introduced to The Livingness:

Tick all that apply - if you don't tick a row we assume that you didn't have that symptom.

	Yes, definitely	Yes, sometimes	No, not much	No, not at all
Ovarian Pain or Cramps	☐	☐	☐	☐
Abdominal Pain	☐	☐	☐	☐
Abdominal Cramps	☐	☐	☐	☐
Endometriosis	☐	☐	☐	☐
Painful periods	☐	☐	☐	☐
Heavy periods	☐	☐	☐	☐
Heavy periods that are worse than before	☐	☐	☐	☐
Heavy periods that last longer than before	☐	☐	☐	☐
Normal periods that last longer than before	☐	☐	☐	☐
Period cycles that are substantially longer than in the past	☐	☐	☐	☐
Irregular period cycles	☐	☐	☐	☐
Very sensitive, tender or sore breasts	☐	☐	☐	☐
Tiredness, worse than in the past	☐	☐	☐	☐
Exhaustion	☐	☐	☐	☐

18. 2 of 3 Perimenopause: **In the 12 months before you attended your first Universal Medicine event** and or were introduced to The Livingness, did you have any of the following:

Tick all that apply - if you don't tick a row we assume that you didn't have that symptom.

	Yes, definitely	Yes, sometimes	No, not much	No, not at all
A desire to have more order in my life	☐	☐	☐	☐
Pre-menstrual tension but few people notice	☐	☐	☐	☐
Pre-menstrual tension noticed by many	☐	☐	☐	☐
Feeling bloated	☐	☐	☐	☐
Hot flushes	☐	☐	☐	☐
Sudden sweats during the day	☐	☐	☐	☐
Feeling relief or appreciation when my periods arrive	☐	☐	☐	☐
Feeling upset or disappointed when my periods arrive	☐	☐	☐	☐
Weight gain	☐	☐	☐	☐
Emotional or sad	☐	☐	☐	☐
Feeling dizzy or faint	☐	☐	☐	☐
Food or drink cravings	☐	☐	☐	☐

Nausea or vomiting	☐	☐	☐	☐

19. **In the last 12 months** did you have any of the following:
('the last 12 months' means the 12 months ending today)
If you had your first Universal Medicine event less than 12 months ago, did you have any of the following since you had your first Universal Medicine event:

Tick all that apply - if you don't tick a row we assume that you didn't have that symptom.

	Yes, definitely	Yes, sometimes	No, not much	No, not at all
A desire to have more order in my life	☐	☐	☐	☐
Pre-menstrual tension but few people notice	☐	☐	☐	☐
Pre-menstrual tension noticed by many	☐	☐	☐	☐
Feeling bloated	☐	☐	☐	☐
Hot flushes	☐	☐	☐	☐
Sudden sweats during the day	☐	☐	☐	☐
Feeling relief or appreciation when my periods arrive	☐	☐	☐	☐
Feeling upset or disappointed when my periods arrive	☐	☐	☐	☐
Weight gain	☐	☐	☐	☐
Crying for no reason	☐	☐	☐	☐
Feeling dizzy or faint	☐	☐	☐	☐
Food or drink cravings	☐	☐	☐	☐
Nausea or vomiting	☐	☐	☐	☐
Trouble breathing	☐	☐	☐	☐

20. 3 of 3: Perimenopause: **In the 12 months before you attended your first Universal Medicine event** and or were introduced to The Livingness, did you have any of the following:

Tick all that apply - if you don't tick a row we assume that you didn't have that symptom.

	Yes, definitely	Yes, sometimes	No, not much	No, not at all
Feeling less fit than before	☐	☐	☐	☐
Feeling uncomfortable in the presence of others more frequently	☐	☐	☐	☐
Difficulty concentrating	☐	☐	☐	☐
Being more forgetful at times	☐	☐	☐	☐
Feeling anxious	☐	☐	☐	☐
Having bursts of energy	☐	☐	☐	☐
Having back aches	☐	☐	☐	☐
Buzzing or ringing in the ears	☐	☐	☐	☐
Mood swings - rapid changes in how you feel	☐	☐	☐	☐
Headaches	☐	☐	☐	☐
Pimples, dry skin, greasy skin, erratic skin in general	☐	☐	☐	☐

21. **In the last 12 months** did you have any of the following:
('the last 12 months' means the 12 months ending today)
If you had your first Universal Medicine event less than 12 months ago, did you have any of the following since you had your first Universal Medicine event:

Tick all that apply - if you don't tick a row we assume that you didn't have that symptom.

	Yes, definitely	Yes, sometimes	No, not much	No, not at all

Feeling uncomfortable in the presence of others more frequently	☐	☐	☐	☐
Difficulty concentrating	☐	☐	☐	☐
Being more forgetful at times	☐	☐	☐	☐
Feeling anxious	☐	☐	☐	☐
Having bursts of energy	☐	☐	☐	☐
Having back aches	☐	☐	☐	☐
Mood swings - rapid changes in how you feel	☐	☐	☐	☐
Headaches	☐	☐	☐	☐
Pimples, dry skin, greasy skin, erratic skin in general	☐	☐	☐	☐

22. How do you feel about the following statements?

	Strongly disagree	Disagree	Neither agree or disagree	Agree	Strongly agree
I find my body is more easily affected by my daily living choices than in the past	○	○	○	○	○
I feel I am at the mercy of the hormonal changes in my body	○	○	○	○	○
I wonder if I am still of worth once I don't have periods any more	○	○	○	○	○
Once I have no periods any more I won't be seen any more	○	○	○	○	○
Losing my periods gives me a feeling of loss and grief	○	○	○	○	○
Perimenopause is a period of grace giving me time to adjust to my new role in the world	○	○	○	○	○
I will be relieved or happy when my period stops	○	○	○	○	○
I feel quite unsettled because my cycle is so irregular	○	○	○	○	○
I find perimenopause very upsetting	○	○	○	○	○
I feel my body is calling me to be more still	○	○	○	○	○
I feel my body is calling me to be more tender	○	○	○	○	○
I take more supplements than before	○	○	○	○	○
I feel steadier than before	○	○	○	○	○
I am nurturing myself more	○	○	○	○	○
I changed my life to a more harmonious pace	○	○	○	○	○
My body shape has changed in a good way	○	○	○	○	○
I feel much wiser	○	○	○	○	○
I feel embarrassed when I suddenly sweat or have hot flushes	○	○	○	○	○

23. How **did you feel** about the following statements **at the time you had your first Universal Medicine event**?

	Strongly disagree	Disagree	Neither agree or disagree	Agree	Strongly agree
I find my body is more easily affected by my daily living choices than in the past	○	○	○	○	○
I feel I am a victim to the hormonal changes in my body	○	○	○	○	○
I wonder if I am still of worth once I don't have periods any more	○	○	○	○	○
Once I have no periods any more I won't be seen any more	○	○	○	○	○
Losing my periods gives me a feeling of loss and grief	○	○	○	○	○
Perimenopause is a period of grace giving me time to adjust to my new role in the world	○	○	○	○	○
I feel quite unsettled because my cycle is so irregular	○	○	○	○	○

I find perimenopause very upsetting					
I feel my body is calling me to be more still					
I feel my body is calling me to be more tender					
I take more supplements than before					
I feel steadier than before					
I am nurturing myself more					
I changed my life to a more harmonious pace					
My body shape has changed in a good way					
I feel much wiser					
I feel embarrassed when I suddenly sweat or have hot flushes					

Women who have periods

24. **In the 12 months before you attended your first Universal Medicine event** and or were introduced to The Livingness, did you have any of the following:

*Tick all that apply - **if you don't tick a row we assume that you didn't have that symptom.***

	Yes, definitely	Yes, sometimes	No, not much	No, not at all
Abdominal Cramps				
Endometriosis				
Painful periods				
Heavy periods				
Skipped periods				
Heavy clotting, slight clotting				
Constipation and other unusual bowel movements				
Swollen breasts				
Very sensitive, tender or sore breasts				
Severe tiredness				
Exhaustion				
Tired or aching legs				

25. **In the last 12 months** did you have any of the following:
('the last 12 months' means the 12 months ending today)
If you attended your first Universal Medicine event less than 12 months ago, did you have any of the following since you attended your first Universal Medicine event or were introduced to The Livingness:

Tick all that apply - if you don't tick a row we assume that you didn't have that symptom.

	Yes, definitely	Yes, sometimes	No, not much	No, not at all
Abdominal Cramps				
Endometriosis				
Painful periods				
Heavy periods				
Skipped periods				
Heavy clotting, slight clotting				
Constipation and other unusual bowel movements				
Swollen breasts				
Very sensitive, tender or sore breasts				

Severe tiredness	☐	☐	☐	☐
Exhaustion	☐	☐	☐	☐
Tired or aching legs	☐	☐	☐	☐

26. Part 2 of 3: **In the 12 months before you attended your first Universal Medicine event** and or were introduced to The Livingness, did you have any of the following:

Tick all that apply - if you don't tick a row we assume that you didn't have that symptom.

	Yes, definitely	Yes, sometimes	No, not much	No, not at all
A desire for everything to be neater and more tidy	☐	☐	☐	☐
Pre-menstrual tension but few people noticed	☐	☐	☐	☐
Pre-menstrual tension noticed by many	☐	☐	☐	☐
Intense irritation with others	☐	☐	☐	☐
Feeling bloated	☐	☐	☐	☐
Feeling bothered or inconvenienced by my periods	☐	☐	☐	☐
Feeling controlled by my periods	☐	☐	☐	☐
Weight gain	☐	☐	☐	☐
Emotional or sad	☐	☐	☐	☐
Feeling dizzy or faint	☐	☐	☐	☐
Food or drink cravings	☐	☐	☐	☐
Nausea or vomiting	☐	☐	☐	☐

27. **In the last 12 months** did you have any of the following:
('the last 12 months' means the 12 months ending today)
If you attended your first Universal Medicine event less than 12 months ago, did you have any of the following since you attended your first Universal Medicine event or were introduced to The Livingness:

Tick all that apply - if you don't tick a row we assume that you didn't have that symptom.

	Yes, definitely	Yes, sometimes	No, not much	No, not at all
A desire for everything to be neater and more tidy	☐	☐	☐	☐
Pre-menstrual tension but few people noticed	☐	☐	☐	☐
Pre-menstrual tension noticed by many	☐	☐	☐	☐
Intense irritation with others	☐	☐	☐	☐
Feeling bloated	☐	☐	☐	☐
Feeling bothered or inconvenienced by my periods	☐	☐	☐	☐
Feeling controlled by my periods	☐	☐	☐	☐
Weight gain	☐	☐	☐	☐
Emotional or sad	☐	☐	☐	☐
Feeling dizzy or faint	☐	☐	☐	☐
Food or drink cravings	☐	☐	☐	☐
Nausea or vomiting	☐	☐	☐	☐

28. Part 3 of 3: **In the 12 months before you attended your first Universal Medicine event** and or were introduced to The Livingness, did you have any of the following:

Tick all that apply - if you don't tick a row we assume that you didn't have that symptom.

	Yes, definitely	Yes, sometimes	No, not much	No, not at all

Feeling uncomfortable in the presence of others more frequently	☐	☐	☐	☐
Difficulty concentrating	☐	☐	☐	☐
Feeling anxious	☐	☐	☐	☐
Having bursts of energy	☐	☐	☐	☐
Having back aches	☐	☐	☐	☐
Aching or sore hips	☐	☐	☐	☐
Mood swings - rapid changes in how you feel	☐	☐	☐	☐
Headaches	☐	☐	☐	☐
Pimples, dry skin, greasy skin, erratic skin in general	☐	☐	☐	☐
An overall feeling of harmony in the body	☐	☐	☐	☐

29. **In the last 12 months** did you have any of the following:
('the last 12 months' means the 12 months ending today)
If you attended your first Universal Medicine event less than 12 months ago, did you have any of the following since you attended your first Universal Medicine event or were introduced to The Livingness:

Tick all that apply - if you don't tick a row we assume that you didn't have that symptom.

	Yes, definitely	Yes, sometimes	No, not much	No, not at all
Feeling uncomfortable in the presence of others more frequently	☐	☐	☐	☐
Difficulty concentrating	☐	☐	☐	☐
Feeling anxious	☐	☐	☐	☐
Having bursts of energy	☐	☐	☐	☐
Having back aches	☐	☐	☐	☐
Aching or sore hips	☐	☐	☐	☐
Mood swings - rapid changes in how you feel	☐	☐	☐	☐
Headaches	☐	☐	☐	☐
Pimples, dry skin, greasy skin, erratic skin in general	☐	☐	☐	☐
An overall feeling of harmony in the body	☐	☐	☐	☐

30. How do you feel about the following statements?

	Strongly disagree	Disagree	Somewhat disagree	Somewhat agree	Agree	Strongly agree
I talk with other women about my period comfortably	○	○	○	○	○	○
I feel at the mercy of the hormonal cycles in my body	○	○	○	○	○	○
I dislike having periods	○	○	○	○	○	○
I am more tired during my period	○	○	○	○	○	○
I often experience a pressure or build up before my period and then relief when my period arrives	○	○	○	○	○	○
Periods are a sign of womanhood	○	○	○	○	○	○
Periods are a good sign of your general health	○	○	○	○	○	○
I pay little attention to my period cycle, it just comes and goes	○	○	○	○	○	○
I notice how much my body supports me in the timing of when my periods arrive	○	○	○	○	○	○
My periods can come at very inconvenient times	○	○	○	○	○	○
I notice how period pain, tender breasts, pimples or food cravings are related to how I am living	○	○	○	○	○	○
I notice how my emotions change during my cycle	○	○	○	○	○	○

I use pain killers to stop period pain so I can get on with my day	◯	◯	◯	◯	◯	◯
I feel a need to be more gentle with myself when menstruating	◯	◯	◯	◯	◯	◯

31. How **did you feel** about the following statements **at the time you had your first Universal Medicine event**?

	Strongly disagree	Disagree	Somewhat disagree	Somewhat agree	Agree	Strongly agree
I talk with other women about my period comfortably	◯	◯	◯	◯	◯	◯
I feel at the mercy of the hormonal cycles in my body	◯	◯	◯	◯	◯	◯
I dislike having periods	◯	◯	◯	◯	◯	◯
I am more tired during my period	◯	◯	◯	◯	◯	◯
I often experience a pressure or build up before my period and then relief when my period arrives	◯	◯	◯	◯	◯	◯
Periods are a sign of womanhood	◯	◯	◯	◯	◯	◯
Periods are a good sign of your general health	◯	◯	◯	◯	◯	◯
I pay little attention to my period cycle, it just comes and goes	◯	◯	◯	◯	◯	◯
I notice how much my body supports me in the timing of when my periods arrive	◯	◯	◯	◯	◯	◯
My periods can come at very inconvenient times	◯	◯	◯	◯	◯	◯
I notice how period pain, tender breasts, pimples or food cravings are related to how I am living	◯	◯	◯	◯	◯	◯
I notice how my emotions change during my cycle	◯	◯	◯	◯	◯	◯
I use pain killers to stop period pain so I can get on with my day	◯	◯	◯	◯	◯	◯
I feel a need to be more gentle with myself when menstruating	◯	◯	◯	◯	◯	◯

The questions on the first half of this page ask only about NOW - how your health is NOW and about how your health limits certain activities NOW.

32. In general, would you say your health is:

◯ Excellent ◯ Very good ◯ Good ◯ Fair ◯ Poor

33. Compared to one year ago, how would you rate your health in general now?

◯ Much better now than one year ago
◯ Somewhat better now than one year ago
◯ About the same now as one year ago
◯ Somewhat worse now than one year ago
◯ Much worse now than one year ago

34. The following questions are about activities you might do during a typical day. Does **your health now limit you** in these activities? If so, how much?

	Yes, limited a lot	Yes, limited a little	No, not limited at all
**Vigorous** activities, such as running, lifting heavy objects, participating in strenuous sports	◯	◯	◯
**Moderate** activities, such as moving a table, pushing a vacuum cleaner, bowling or playing golf	◯	◯	◯
Lifting or carrying groceries	◯	◯	◯
Climbing **several** flights of stairs	◯	◯	◯
Climbing **one** flight of stairs	◯	◯	◯

Bending, kneeling or stooping	◯	◯	◯
Walking **more than one** kilometre	◯	◯	◯
Walking **half** a kilometre	◯	◯	◯
Walking 100 metres	◯	◯	◯
Bathing or dressing yourself	◯	◯	◯

The following questions ask about your health
IN THE LAST FOUR WEEKS.

35. During the **past four weeks**, have you had any of the following problems with your work (including your work outside the home and housework) or other regular daily activities
as a result of your physical health?

	Yes	No
Cut down on the amount of time you spent on work or other activities	◯	◯
Accomplished less than you would like	◯	◯
Were limited in the kind of work or other activities	◯	◯
Had difficulty performing the work or other activities (eg it took extra effort)	◯	◯

36. During the **past four weeks**, have you had any of the following problems with your work or other regular daily activities **as a result of any emotional problems**
(such as feeling depressed or anxious)?

	Yes	No
Cut down on the amount of time you spent on work or other activities	◯	◯
Accomplished less than you would like	◯	◯
Didn't do work or other activities as carefully as usual	◯	◯

37. During the **past four weeks**, to what extent have your **physical health or emotional problems** interfered with your normal social activities with family, friends,
neighbours or groups?

◯ Not at all ◯ Slightly ◯ Moderately ◯ Quite a bit ◯ Extremely

38. How much **bodily** pain have you had during the **past four weeks**?

◯ No bodily pain
◯ Very mild
◯ Mild
◯ Moderate
◯ Severe
◯ Very severe

39. During the **past four weeks**, how much did **pain** interfere with your normal work
(including both work outside the home and housework)?

◯ Not at all ◯ A little bit ◯ Moderately ◯ Quite a bit ◯ Extremely

40. For each question, please give the one answer that comes closest to the way you have
been feeling. How much of the time during the **past four weeks**:

	All of the time	Most of the time	A good bit of the time	Some of the time	A little of the time	None of the time
Did you feel full of life?	◯	◯	◯	◯	◯	◯
Have you been a very nervous person?	◯	◯	◯	◯	◯	◯
Have you felt so down in the dumps that nothing						

could cheer you up?	◯	◯	◯	◯	◯	◯
Have you felt calm and peaceful?	◯	◯	◯	◯	◯	◯
Did you have a lot of energy?	◯	◯	◯	◯	◯	◯
Have you felt down?	◯	◯	◯	◯	◯	◯
Did you feel worn out?	◯	◯	◯	◯	◯	◯
Have you been a happy person?	◯	◯	◯	◯	◯	◯
Did you feel tired?	◯	◯	◯	◯	◯	◯

41. During the **past four weeks**, how much of the time have your **physical health or emotional problems** interfered with your social activities (like visiting friends, relatives, etc)?

◯ All of the time
◯ Most of the time
◯ Some of the time
◯ A little of the time
◯ None of the time

We are now asking the same questions again but for the time just before you had your first Universal Medicine event. If you don't remember, simply move to the next question. If you DO remember, it would be very helpful if you answer the question again.

42. At the time you attended your first Universal Medicine event or were introduced to The Livingness, in general, would you say your health was:

◯ Excellent ◯ Very good ◯ Good ◯ Fair ◯ Poor

43. How would you rate your health in general at the time you had your first Universal Medicine event?

◯ Much better at the time of my first Universal Medicine event than one year before my first UM event
◯ Somewhat better at the time of my first Universal Medicine event than one year before then
◯ About the same at the time of my first Universal Medicine event as one year before then
◯ Somewhat worse at the time of my first Universal Medicine event than one year before then
◯ Much worse at the time of my first Universal Medicine event than one year before then

44. The following questions are about activities you might have done during a typical day. At the time you attended your first Universal Medicine event or were introduced to The Livingness, did **your health *then* limit you** in these activities? If so, how much?

	Yes, limited a lot	Yes, limited a little	No, not limited at all
**Vigorous** activities, such as running, lifting heavy objects, participating in strenuous sports	◯	◯	◯
**Moderate** activities, such as moving a table, pushing a vacuum cleaner, bowling or playing golf	◯	◯	◯
Lifting or carrying groceries	◯	◯	◯
Climbing **several** flights of stairs	◯	◯	◯
Climbing **one** flights of stairs	◯	◯	◯
Bending, kneeling or stooping	◯	◯	◯
Walking **more than one** kilometre	◯	◯	◯
Walking **half** a kilometre	◯	◯	◯
Walking 100 metres	◯	◯	◯
Bathing or dressing yourself	◯	◯	◯

The questions on this page and the next one ask about your health

IN THE FOUR WEEKS BEFORE YOU attended your first Universal Medicine event or were introduced to The Livingness.

45. In the **four weeks before** you attended your first Universal Medicine event or were introduced to The Livingness, have you had any of the following problems with your work (including your work outside the home and housework) or other regular daily activities **as a result of your physical health**?

	Yes	No
Cut down on the amount of time you spent on work or other activities	◯	◯
Accomplished less than you would like	◯	◯
Were limited in the kind of work or other activities	◯	◯
Had difficulty performing the work or other activities (eg it took extra effort)	◯	◯

46. In the **four weeks before** you attended your first Universal Medicine event or were introduced to The Livingness, have you had any of the following problems with your work or other regular daily activities **as a result of any emotional problems** (such as feeling depressed or anxious)?

	Yes	No
Cut down on the amount of time you spent on work or other activities	◯	◯
Accomplished less than you would like	◯	◯
Didn’t do work or other activities as carefully as usual	◯	◯

47. In the **four weeks before** you attended your first Universal Medicine event or were introduced to The Livingness, to what extent did your **physical health or emotional problems** interfere with your normal social activities with family, friends, neighbours or groups?

◯ Not at all ◯ Slightly ◯ Moderately ◯ Quite a bit ◯ Extremely

48. How much **bodily** pain have you had in the **four weeks before** you attended your first Universal Medicine event or were introduced to The Livingness?

◯ No bodily pain
◯ Very mild
◯ Mild
◯ Moderate
◯ Severe
◯ Very severe

49. In the **four weeks before** you attended your first Universal Medicine event or were introduced to The Livingness, how much did **pain interfere with your normal work (including both work outside the home and housework)?**

◯ Not at all ◯ A little bit ◯ Moderately ◯ Quite a bit ◯ Extremely

50. For each question, please give the one answer that comes closest to the way you have
been feeling. How much of the time In the **four weeks before** you attended your first Universal Medicine event or were introduced to The Livingness:

	All of the time	Most of the time	A good bit of the time	Some of the time	A little of the time	None of the time
Did you feel full of life?	◯	◯	◯	◯	◯	◯
Have you been a very nervous person?	◯	◯	◯	◯	◯	◯
Have you felt so down in the dumps that nothing could cheer you up?	◯	◯	◯	◯	◯	◯
Have you felt calm and peaceful?	◯	◯	◯	◯	◯	◯
Did you have a lot of energy?	◯	◯	◯	◯	◯	◯
Have you felt down?	◯	◯	◯	◯	◯	◯
Did you feel worn out?	◯	◯	◯	◯	◯	◯
Have you been a happy person?	◯	◯	◯	◯	◯	◯
Did you feel tired?	◯	◯	◯	◯	◯	◯

51. In the **four weeks before** you attended your first Universal Medicine event or were introduced to The Livingness, how much of the time have your **physical health or emotional problems** interfered with your social activities (like visiting friends, relatives, etc)?

- All of the time
- Most of the time
- Some of the time
- A little of the time
- None of the time

More than half way

Thank you for getting this far. The next questions will support us **a lot** to see how Women in Livingness compare to other women who answered the Australian Longitudinal Study into Women's Health (www.alswh.org).

Feel free to take a break and to continue later. The survey will continue where you left off even if you turned off your computer or tablet or phone in-between.
If by any chance you can't resume or if you have any other difficulty, email cs@inyourinterest.com.au and Christoph will support you.

52. How **true** or **false** is **each** of the following statements for you?

	Definitely true	Mostly true	Don't know	Mostly false	Definitely false
I seem to get sick a little easier than other people	◯	◯	◯	◯	◯
I am as healthy as anybody I know	◯	◯	◯	◯	◯
I expect my health to get worse	◯	◯	◯	◯	◯
My health is excellent	◯	◯	◯	◯	◯

53. At the **time you attended your first Universal Medicine event** or were introduced to The Livingness, how **true** or **false** was **each** of the following statements for you?

	Definitely true	Mostly true	Don't know	Mostly false	Definitely false
I seemed to get sick a little easier than other people	◯	◯	◯	◯	◯
I was as healthy as anybody I know	◯	◯	◯	◯	◯
I expected my health to get worse	◯	◯	◯	◯	◯
My health was excellent	◯	◯	◯	◯	◯

54. How many times have you consulted the following people for **your own health** in the **last twelve months**?

	None	Once or twice	3 or 4 times	5 or 6 times	7-12 times	13-24 times	25 or more times
A family doctor or another General Practitioner (GP)	◯	◯	◯	◯	◯	◯	◯
A hospital doctor (eg in outpatients or casualty)	◯	◯	◯	◯	◯	◯	◯
A specialist doctor	◯	◯	◯	◯	◯	◯	◯

55. In the **twelve months before you attended your first Universal Medicine event** or were introduced to The Livingness, how many times have you consulted the following people for **your own health**?

	None	Once or twice	3 or 4 times	5 or 6 times	7-12 times	13-24 times	25 or more times
A family doctor or another General Practitioner (GP)	◯	◯	◯	◯	◯	◯	◯
A hospital doctor (eg in outpatients or casualty)	◯	◯	◯	◯	◯	◯	◯

A specialist doctor	○	○	○	○	○	○	○

56. Have you consulted the following people for **your own health** in the **last twelve months**?

	Yes	No
Physiotherapist	○	○
Counsellor / Psychologist / Social worker	○	○
A community nurse, practice nurse, or nurse practitioner	○	○
Optician / Optometrist	○	○
Dietitian	○	○
Podiatrist	○	○
Massage therapist	○	○
Naturopath / Herbalist	○	○
Chiropractor	○	○
Osteopath	○	○
Acupuncturist	○	○
Other alternative health practitioner (eg aromatherapist, homeopath, reflexologist, iridologist)	○	○
An Esoteric Practitioner	○	○

57. In the **twelve months before you attended your first Universal Medicine event** or were introduced to The Livingness, did you consult the following people for **your own health**?

	Yes	No
Physiotherapist	○	○
Counsellor / Psychologist / Social worker	○	○
A community nurse, practice nurse, or nurse practitioner	○	○
Optician / Optometrist	○	○
Dietitian	○	○
Podiatrist	○	○
Massage therapist	○	○
Naturopath / Herbalist	○	○
Chiropractor	○	○
Osteopath	○	○
Acupuncturist	○	○
Other alternative health practitioner (eg aromatherapist, homeopath, reflexologist, iridologist)	○	○
An Esoteric Practitioner	○	○

58. How often have you used the following therapies for **your own health** in the **last twelve months**?

	Never	Rarely	Sometimes	Often
Vitamins / Minerals	○	○	○	○
Yoga or meditation	○	○	○	○
Herbal medicines	○	○	○	○
Aromatherapy oils	○	○	○	○
Chinese medicines	○	○	○	○
Prayer or spiritual healing	○	○	○	○
An esoteric therapy	○	○	○	○

Other alternative therapies	◯	◯	◯	◯

59. In the **twelve months before you attended your first Universal Medicine event** or were introduced to The Livingness, how often have you used the following therapies for **your own health**?

	Never	Rarely	Sometimes	Often
Vitamins / Minerals	◯	◯	◯	◯
Yoga or meditation	◯	◯	◯	◯
Herbal medicines	◯	◯	◯	◯
Aromatherapy oils	◯	◯	◯	◯
Chinese medicines	◯	◯	◯	◯
Prayer or spiritual healing	◯	◯	◯	◯
An esoteric therapy	◯	◯	◯	◯
Other alternative therapies	◯	◯	◯	◯

60. Have you **ever** had an abnormal result from:

	Yes	No	Don't know
A Pap test?	◯	◯	◯
A mammogram?	◯	◯	◯

61. Are you currently taking:

	Yes	No
The oral contraceptive pill?	◯	◯
Hormone Replacement Therapy (HRT)?	◯	◯

62. Have you:

	Yes	No
Had a hysterectomy?	◯	◯
Had a period or menstrual bleeding in the last 12 months?	◯	◯
Had a period or menstrual bleeding in the last 3 months?	◯	◯

63. At the **time you attended your first Universal Medicine event** or were introduced to The Livingness, Have you

	Yes	No
Had a period or menstrual bleeding in the previous 12 months?	◯	◯
Had a period or menstrual bleeding in the previous 3 months?	◯	◯

64. Compared with twelve months ago, are your periods:

◯ Less frequent ◯ About the same ◯ More frequent ◯ Changeable

65. In the **12 months before you attended your first Universal Medicine event** or were introduced to The Livingness, were your periods (becoming):

◯ Less frequent ◯ About the same ◯ More frequent ◯ Changeable

66. At what age did you have your first menstrual period?
(Age in years, for example: 12)

67. If you have reached menopause, at what age did your periods completely stop?
(Age in years)

68. How would you rate the overall condition of your teeth, dentures or gums?

○ Excellent ○ Very good ○ Good ○ Fair ○ Poor

69. At the **time you attended your first Universal Medicine event** or were introduced to The Livingness, how would you have rated the overall condition of your teeth, dentures or gums?

○ Excellent ○ Very good ○ Good ○ Fair ○ Poor

70. Have you ever been diagnosed or treated for:

	Yes
Insulin dependent (Type 1) diabetes	☐
Non-insulin dependent (Type 2) diabetes	☐
Hyperthyroidism (Overly active thyroid)	☐
Hypothyroidism (Under active thyroid)	☐
Impaired glucose tolerance	☐
Osteoarthritis	☐
Rheumatoid arthritis	☐
Other arthritis	☐
Heart disease (including heart attack, angina)	☐
Hypertension (high blood pressure)	☐
Thrombosis	☐
Stroke	☐
Low iron level (iron deficiency or anaemia)	☐
Asthma	☐
Bronchitis / emphysema	☐
Osteoporosis	☐
Endometriosis	☐
Polycystic Ovary Syndrome	☐
Breast cancer	☐
Cervical cancer	☐
Skin cancer (including melanoma)	☐
Other cancer	☐
Depression	☐
Anxiety / nervous disorder	☐
Other psychiatric disorder	☐
Chronic Fatigue Syndrome	☐
Sexually transmitted infection (eg genital herpes or warts, chlamydia)	☐
Polyps or Fibroids related to female organs	☐
None of these conditions	☐

71. Have you ever had any of the following operations or procedures

	Yes
A non-cancerous cyst removed	☐
Polyps or fibroids related to female organs removed	☐
Thyroid removed	☐
One but not both ovaries removed	☐
Both ovaries removed	☐
Repair of prolapsed vagina, bladder or bowel?	☐
Endometrial ablation (removal of the lining of the uterus)	☐
Joint replacement (eg hip, knee)	☐
Mastectomy (removal of one or both breasts)	☐
Lumpectomy (removal of lump from breasts)	☐
Removal of skin cancer	☐
Any cancer surgery (other than skin or breast)	☐
Chemotherapy or radiotherapy for any cancer	☐
Breast biopsy (taking a sample of breast tissue)	☐
Hysteroscopy (investigative procedure to examine the uterus)	☐
Cholecystectomy (gall bladder removed)	☐
Gastroscopy / colonoscopy	☐
None of these	☐

72. Do you have any of these sleeping problems?

	Yes
Waking up in the early hours of the morning	☐
Lying awake for most of the night	☐
Taking a long time to get to sleep	☐
Worry keeping you awake at night	☐
Sleeping badly at night	☐
None of these problems	☐

73. At the **time you attended your first Universal Medicine event** or were introduced to The Livingness, did you have any of these sleeping problems?

	Yes
Waking up in the early hours of the morning	☐
Lying awake for most of the night	☐
Taking a long time to get to sleep	☐
Worry keeping you awake at night	☐
Sleeping badly at night	☐
None of these problems	☐

74. In the **last twelve months**, have you had any of the following:

	Never	Rarely	Sometimes	Often
Allergies, hayfever, sinusitis	○	○	○	○
Breathing difficulty	○	○	○	○
Indigestion / heartburn	○	○	○	○

Chest pain	○	○	○	○
Headaches / migraines	○	○	○	○
Severe tiredness	○	○	○	○
Stiff or painful joints	○	○	○	○
Back pain	○	○	○	○
Urine that burns or stings	○	○	○	○
Haemorrhoids (piles)	○	○	○	○
Other bowel problems	○	○	○	○
Vaginal irritation or unusual discharge	○	○	○	○
Hot flushes	○	○	○	○
Night sweats	○	○	○	○
Eyesight problems	○	○	○	○
Leaking urine	○	○	○	○
Mouth, teeth or gum problems	○	○	○	○
Avoided eating some foods because of problems with your teeth, mouth or dentures	○	○	○	○
Toothache	○	○	○	○
Hearing problems	○	○	○	○
Depression	○	○	○	○
Anxiety	○	○	○	○
Episodes of intense anxiety (eg panic attacks)	○	○	○	○
Palpitations (feeling that your heart is racing or fluttering in your chest)	○	○	○	○

75. In the **twelve months before you attended your first Universal Medicine event** or were introduced to The Livingness, have you had any of the following:

	Never	Rarely	Sometimes	Often
Allergies, hayfever, sinusitis	○	○	○	○
Breathing difficulty	○	○	○	○
Indigestion / heartburn	○	○	○	○
Chest pain	○	○	○	○
Headaches / migraines	○	○	○	○
Severe tiredness	○	○	○	○
Stiff or painful joints	○	○	○	○
Back pain	○	○	○	○
Urine that burns or stings	○	○	○	○
Haemorrhoids (piles)	○	○	○	○
Other bowel problems	○	○	○	○
Vaginal irritation or unusual discharge	○	○	○	○
Hot flushes	○	○	○	○
Night sweats	○	○	○	○
Eyesight problems	○	○	○	○
Leaking urine	○	○	○	○
Mouth, teeth or gum problems	○	○	○	○
Avoided eating some foods because of problems with your teeth, mouth or dentures	○	○	○	○
Toothache	○	○	○	○
Hearing problems	○	○	○	○

Depression	○	○	○	○
Anxiety	○	○	○	○
Episodes of intense anxiety (eg panic attacks)	○	○	○	○
Palpitations (feeling that your heart is racing or fluttering in your chest)	○	○	○	○

76. Over the **last twelve months**, how stressed have you felt about the following areas of your life:

	Not applicable	Not at all stressed	Somewhat stressed	Moderately stressed	Very stressed	Extremely stressed
Own health	○	○	○	○	○	○
Health of family members	○	○	○	○	○	○
Work / Employment	○	○	○	○	○	○
Living arrangements	○	○	○	○	○	○
Study	○	○	○	○	○	○
Money	○	○	○	○	○	○
Relationship with parents	○	○	○	○	○	○
Relationship with partner / spouse	○	○	○	○	○	○
Relationship with children	○	○	○	○	○	○
Relationship with other family members	○	○	○	○	○	○

77. Over the **twelve months before you attended your first Universal Medicine event** or were introduced to The Livingness, how stressed did you feel about the following areas of your life:

	Not applicable	Not at all stressed	Somewhat stressed	Moderately stressed	Very stressed	Extremely stressed
Own health	○	○	○	○	○	○
Health of family members	○	○	○	○	○	○
Work / Employment	○	○	○	○	○	○
Living arrangements	○	○	○	○	○	○
Study	○	○	○	○	○	○
Money	○	○	○	○	○	○
Relationship with parents	○	○	○	○	○	○
Relationship with partner / spouse	○	○	○	○	○	○
Relationship with children	○	○	○	○	○	○
Relationship with other family members	○	○	○	○	○	○

78. How much do you agree or disagree with each of the following statements?

	Disagree strongly	Disagree	Disagree slightly	Agree slightly	Agree	Agree strongly
At home, I feel I have control over what happens in most situations	○	○	○	○	○	○
I feel that what happens in my life is often determined by factors beyond my control	○	○	○	○	○	○
Over the next 5-10 years I expect to have more positive than negative experiences	○	○	○	○	○	○

I often have the feeling that I am being treated unfairly	○	○	○	○	○	○
In the past 10 years my life has been full of changes without my knowing what will happen next	○	○	○	○	○	○
I gave up trying to make big improvements or changes in my life a long time ago	○	○	○	○	○	○

79. At the **time you attended your first Universal Medicine event** or were introduced to The Livingness, how much did you agree or disagree with each of the following statements?

	Disagree strongly	Disagree	Disagree slightly	Agree slightly	Agree	Agree strongly
At home, I felt I had control over what happens in most situations	○	○	○	○	○	○
I felt that what happens in my life was often determined by factors beyond my control	○	○	○	○	○	○
Over the following 5-10 years I expected to have more positive than negative experiences	○	○	○	○	○	○
I often had the feeling that I was being treated unfairly	○	○	○	○	○	○
In the previous 10 years my life had been full of changes without my knowing what would happen next	○	○	○	○	○	○
I gave up trying to make big improvements or changes in my life a long time ago	○	○	○	○	○	○

80. Below is a list of the ways you might have felt or behaved. Please indicate how often you have felt this way **during the last week**.

	Rarely or none of the time (less than 1 day)	Some or a little of the time (1-2 days)	Occasionally or a moderate amount of the time (3-4 days)	Most or all of the time (5-7 days)
I was bothered by things that don't usually bother me	○	○	○	○
I had trouble keeping my mind on what I was doing	○	○	○	○
I felt depressed	○	○	○	○
I felt that everything I did was an effort	○	○	○	○
I felt hopeful about the future	○	○	○	○
I felt fearful	○	○	○	○
My sleep was restless	○	○	○	○
I was happy	○	○	○	○
I felt lonely	○	○	○	○
I could not "get going"	○	○	○	○
I felt terrific	○	○	○	○

81. Below is the same list of the ways you might have felt or behaved. Please indicate how often you have felt this way **in the week before you attended your first Universal Medicine event** or were introduced to The Livingness.

	Rarely or none of the time (less than 1 day)	Some or a little of the time (1-2 days)	Occasionally or a moderate amount of the time (3-4 days)	Most or all of the time (5-7 days)
I was bothered by things that don't usually bother me	○	○	○	○
I had trouble keeping my mind on what I was doing	○	○	○	○
I felt depressed	○	○	○	○

I felt that everything I did was an effort				
I felt hopeful about the future				
I felt fearful				
My sleep was restless				
I was happy				
I felt lonely				
I could not "get going"				
I felt terrific				

82. The following ten questions ask about how you have been feeling in the **last 30 days.** For each question, tick the option that best describes the amount of time you felt that way.

During that month, how often did you feel ...

	None of the time	A little of the time	Some of the time	Most of the time	All of the time
... feel tired out for no good reason?					
... nervous?					
... so nervous that nothing could calm you down?					
... hopeless?					
... restless or fidgety?					
... so restless you could not sit still?					
... depressed?					
... that everything was an effort?					
... so sad that nothing could cheer you up?					
... worthless?					

83. In the month **before you attended your first Universal Medicine event or were introduced to The Livingness:**
The following ten questions ask about how you have been feeling in the **last 30 days.** For each question, tick the option that best describes how often you had this feeling.

In those 30 days, how often did you feel ...

	None of the time	A little of the time	Some of the time	Most of the time	All of the time
... tired out for no good reason?					
... nervous?					
... so nervous that nothing could calm you down?					
... hopeless?					
... restless or fidgety?					
... so restless you could not sit still?					
... depressed?					
... that everything was an effort?					
... so sad that nothing could cheer you up?					
... worthless?					

The following sections are about other health habits, time use and your relationships.
Often there are no “right” or “wrong” answers – we are interested only in your opinion or feelings.
If you feel uncomfortable about answering a question, just leave it and go on to the next one, but please try to finish the survey if you can.

84. How much do you weigh in kg? (no clothes or shoes)

kg

85. Alternatively, how much do you weigh in Stones and Pounds (or just in Pounds)

Stones

Pounds

86. At the time **you attended your first Universal Medicine event or were introduced to The Livingness**, how much did you weigh in kg? (no clothes or shoes)

kg

87. Copy of Alternatively, how much **did** you weigh in Stones and Pounds (or just in Pounds)

Stones

Pounds

88. How tall are you without shoes (answer here in cm or in the next question in feet and inches)

cm

89. How tall are you without shoes (answer here in feet and inches if you haven't answered in cm)

feet

inches

90. Have you ever:

	Yes	No
Lost 5 kg (11 pounds) or more on purpose?	○	○
Lost 5 kg or more without any effort?	○	○
Lost 5 kg or more for any other reason?	○	○
Gained 5 kg or more?	○	○

91. Have you used any of these methods to lose weight or to control your weight or shape in the **last twelve months**?

	Yes	No
Commercial weight loss programs (eg Weight Watchers, Lite n' Easy, Sureslim, Jenny Craig)	○	○
Meal replacements or slimming products (eg OPTIFAST, Herbalife)	○	○

Exercise	◯	◯
Cut down on the size of meals or between meal snacks	◯	◯
Cut down on fats (low fat) and / or sugars	◯	◯
Low glycaemic index (GI) diet	◯	◯
Diet book diets (eg Atkins, Zone, CSIRO diet, Liver Cleansing diet)	◯	◯
Laxatives, diuretics or diet pills (eg Xenical, Reductil)	◯	◯
Fasting	◯	◯
Smoking	◯	◯
Other	◯	◯

92. Did you use any of these methods to lose weight or to control your weight or shape in the **twelve months before you attended your first Universal Medicine event** or were introduced to The Livingness?

	Yes	No
Commercial weight loss programs (eg Weight Watchers, Lite n' Easy, Sureslim, Jenny Craig)	◯	◯
Meal replacements or slimming products (eg OPTIFAST, Herbalife)	◯	◯
Exercise	◯	◯
Cut down on the size of meals or between meal snacks	◯	◯
Cut down on fats (low fat) and / or sugars	◯	◯
Low glycaemic index (GI) diet	◯	◯
Diet book diets (eg Atkins, Zone, CSIRO diet, Liver Cleansing diet)	◯	◯
Laxatives, diuretics or diet pills (eg Xenical, Reductil)	◯	◯
Fasting	◯	◯
Smoking	◯	◯
Other	◯	◯

93. The next question is about your alcohol consumption during different stages of your life.
On average, how many drinks did you usually drink **per week** in your:

	Not applicable (I haven't reached this age)	No alcohol	1-7 drinks	8-14 drinks	15 or more drinks
Late teens	◯	◯	◯	◯	◯
20s	◯	◯	◯	◯	◯
30s	◯	◯	◯	◯	◯
40s	◯	◯	◯	◯	◯
50s	◯	◯	◯	◯	◯
60s	◯	◯	◯	◯	◯
70s or later	◯	◯	◯	◯	◯
Last 12 months	◯	◯	◯	◯	◯

94. This question is about your alcohol consumption in **the twelve months before you attended your first Universal Medicine event** or were introduced to The Livingness:

	No alcohol	1-7 drinks	8-14 drinks	15 or more drinks
12 months before your first Universal Medicine event	◯	◯	◯	◯

95. What is the highest level of education you have completed?
(Mark one only)

- ◯ Year 10 or below (for example GCSE or Mittlere Reife)
- ◯ Year 11 or equivalent
- ◯ Year 12 or A-levels or Matriculation (Abitur) equivalent
- ◯ Certificate I / II
- ◯ Certificate III / IV
- ◯ Advanced Diploma / Diploma
- ◯ Bachelor degree
- ◯ Graduate diploma / Graduate certificate
- ◯ Postgraduate degree
- ◯ PhD / Doctorate

Last page

96. How often do you currently smoke cigarettes or any tobacco products?

- ◯ Daily
- ◯ At least weekly (but not daily)
- ◯ Less often than weekly
- ◯ Not at all

97. At the **time you attended your first Universal Medicine event** or were introduced to The Livingness, how often did you smoke cigarettes or any tobacco products?

- ◯ Daily
- ◯ At least weekly (but not daily)
- ◯ Less often than weekly
- ◯ Not at all

98. In a **usual week**, how much time in total do you spend doing the following things?

	I don't do this activity	1- 15 hrs	16- 24 hrs	25- 34 hrs	35- 40 hrs	41- 48 hrs	49 hours or more
Full time paid work	◯	◯	◯	◯	◯	◯	◯
Part-time paid work	◯	◯	◯	◯	◯	◯	◯
Casual paid work	◯	◯	◯	◯	◯	◯	◯
Home duties (own / family home)	◯	◯	◯	◯	◯	◯	◯
Work without pay (eg family business)	◯	◯	◯	◯	◯	◯	◯
Looking for work	◯	◯	◯	◯	◯	◯	◯
Unpaid voluntary work	◯	◯	◯	◯	◯	◯	◯
Active leisure (eg walking, exercise, sport)	◯	◯	◯	◯	◯	◯	◯
Passive leisure (eg TV, music, reading, relaxing)	◯	◯	◯	◯	◯	◯	◯
Studying	◯	◯	◯	◯	◯	◯	◯

99. At the **time you attended your first Universal Medicine event** or were introduced to The Livingness, in a **usual week**, how much time in total did you spend doing the following things?

	I don't do this activity	1- 15 hrs	16- 24 hrs	25- 34 hrs	35- 40 hrs	41- 48 hrs	49 hours or more
Full time paid work	◯	◯	◯	◯	◯	◯	◯
Part-time paid work	◯	◯	◯	◯	◯	◯	◯
Casual paid work	◯	◯	◯	◯	◯	◯	◯
Home duties (own / family home)	◯	◯	◯	◯	◯	◯	◯

Work without pay (eg family business)							
Looking for work							
Unpaid voluntary work							
Active leisure (eg walking, exercise, sport)							
Passive leisure (eg TV, music, reading, relaxing)							
Studying							

100. Managing time is often difficult. How often do you feel:

	Every day	A few times a week	About once a week	About once a month	Never
That you are rushed, pressured, too busy?					
That you have time on your hands that you don't know what to with?					

101. At the **time you attended your first Universal Medicine event** or were introduced to The Livingness, how often did you feel:

	Every day	A few times a week	About once a week	About once a month	Never
That you were rushed, pressured, too busy?					
That you had time on your hands that you didn't know what to with?					

A last question about what food you have **at the moment**:

102. **Food**
On average I have this item:

	More than once a day	Once a day	1-6 times a week	Less than once a week	Not for the past twelve months	Not for the past five years
Coffee, tea, energy drinks or anything with **caffeine**						
Items with added or concentrated **sugar** in it (sweets, mints, cakes, biscuits, chewing gum, ice cream, flavoured yoghurt, healthbars, chocolate, dried fruit, lemonade or soda, coffee, tea or other)						
**Salty** foods (crisps, salted nuts or similar)						
**Dairy** (cheese, yoghurt, ice cream, milk, butter, cream even if part of another dish like cheeseburger or many cakes)						
**Alcohol** (beer, wine, spirits)						

Click on 'next page' below to finish.

We greatly appreciate your time in completing this survey

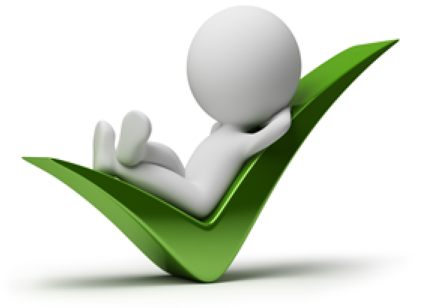

Supplement: Multimedia Appendix 2 [file resprot_v6i11e234_app2.pdf]
